# USP21-EGFR signaling axis is functionally implicated in metastatic colorectal cancer

**DOI:** 10.1038/s41420-024-02255-1

**Published:** 2024-12-18

**Authors:** Ji Hye Shin, Mi-Jeong Kim, Ji Young Kim, Bongkum Choi, Yeeun Kang, Seo Hyun Kim, Ha-Jeong Lee, Dohee Kwon, Yong Beom Cho, Kyeong Kyu Kim, Eunyoung Chun, Ki-Young Lee

**Affiliations:** 1 Department of Immunology, Samsung Biomedical Research Institute, Sungkyunkwan University School of Medicine, Suwon, Republic of Korea; 2 Department of Medicine, Sungkyunkwan University School of Medicine, Suwon, Republic of Korea; 3 Bioanalysis Center, GenNBio Inc., Seongnam, Republic of Korea; 4 Department of Surgery, Samsung Medical Center, Sungkyunkwan University School of Medicine, Seoul, Republic of Korea; 5 Samsung Medical Center, Department of Health Science and Technology, Samsung Advanced Institute for Health Science and Technology, Sungkyunkwan University School of Medicine, Seoul, Republic of Korea; 6 Department of Metabiohealth, Sungkyun Convergence Institute, Sungkyunkwan University, Suwon, Republic of Korea; 7 Research and Development Center, CHA Vaccine Institute, Seongnam, Republic of Korea

**Keywords:** Colorectal cancer, Colon cancer

## Abstract

The emerging role of ubiquitin-specific peptidase 21 (USP21) in stabilizing Fra-1 (FOSL1) highlights its involvement in promoting colorectal cancer (CRC) metastasis. Additionally, a reciprocal link between EGFR signaling and Fra-1 activation has been identified, mediated through matrix metalloproteinases (MMPs). However, the functional implications of the USP21-EGFR signaling axis in metastatic CRC (mCRC) are not fully understood. To investigate the clinical correlation between USP21 and EGFR expression, RNA-Seq data from tumor tissues (*n* = 27) and matched normal tissues (*n* = 27) of 27 mCRC patients were analyzed. Functional studies were performed, including the use of CRISPR/Cas9 to generate *USP21*-knockout (*USP21*-KO) CRC cells, in vitro assays for cancer progression and tumor formation, in vivo xenograft assays in NSG mice. Additionally, the therapeutic effect of the USP21 inhibitor, BAY-805, was evaluated. We found that elevated levels of USP21 and EGFR expression in mCRC patients were associated with poorer survival outcomes. Mechanistically, USP21 was found to enhance EGFR stability by deubiquitinating EGFR, leading to reduced EGFR degradation. *USP21*-KO colon cancer cells exhibited significantly reduced proliferation, migration, colony formation, and 3D tumor spheroid formation in response to EGF. Furthermore, the tumorigenic activity in vivo was markedly diminished in NSG mice xenografted with *USP21-KO* colon cancer cells. Importantly, BAY-805 demonstrated a notable inhibitory effect on the formation of 3D tumor spheroids in colorectal cancer cells stimulated with EGF. These findings suggest that USP21 could be a valuable therapeutic target and predictive biomarker for managing mCRC driven by EGF.

## Introduction

Colorectal cancer (CRC) is a prevalent and lethal malignancy worldwide, influenced by various factors such as lifestyle choices, environmental exposures, viral infections, and smoking [1–3]. Recent advancements in omics data analysis of CRC patients have revealed a wealth of genetic information essential for understanding the pathophysiology of CRC progression and developing targeted therapeutic interventions [4, 5]. The dysregulation of epidermal growth factor receptor (EGFR)-mediated signaling pathways is a significant driver of CRC initiation and progression [2]. EGFR, a key member of the ErbB protein family, plays a critical role in regulating CRC cellular processes, including proliferation, angiogenesis, migration, invasion, and tumorigenicity [2, 6]. Although the precise molecular and cellular mechanisms underlying EGFR upregulation in CRC remain incompletely understood, omics data from CRC patient cohorts consistently show elevated EGFR expression levels in tumor tissues, which correlate with poor prognosis [7].

The regulation of cellular EGFR expression and activation depends on complex EGFR trafficking pathways, which significantly influence the biological outcomes of EGFR signaling in cancer [8]. EGFR’s fate is determined by two main pathways: the recycling pathway and the degradative multivesicular bodies (MVBs)-lysosome pathway [8–10]. Various cellular factors, such as Rab4, Rab35, calcium-modulating cyclophilin ligand (CAML), and Eps15S, are crucial in the EGFR recycling pathway, facilitating prolonged EGFR signaling [8–10]. Ubiquitin-specific peptidases (USPs) have emerged as key regulators of EGFR signaling, with several USP family members involved in modulating EGFR degradation [11–17]. Among these, USP21 has gained attention for stabilizing Fra-1 and programmed death-ligand 1 (PD-L1), thus promoting CRC metastasis [18–22]. Considering the interconnectedness between EGFR signaling, Fra-1 activation, and PD-L1 expression [18, 22], targeting USP21 presents a promising strategy for combating metastatic CRC driven by EGFR signaling. However, the functional and clinical significance of USP21 in metastatic CRC, especially concerning EGFR expression, remains largely unexplored.

This study aims to elucidate the relationship between USP21 and EGFR in metastatic CRC, examining their clinical significance and functional implications. Through comprehensive biochemical and functional analyses, we demonstrate that USP21 plays a critical role in stabilizing EGFR and promoting CRC progression in response to EGF. Conversely, knockout of USP21 (*USP21*-KO) in CRC cells impairs cancer progression and reduces tumor formation following EGF stimulation. Notably, pharmacological inhibition of USP21 with BAY-805 markedly suppresses CRC tumor spheroid formation, highlighting the therapeutic potential of targeting USP21 in mCRC. Collectively, our findings emphasize USP21’s potential as both a therapeutic target and a predictive biomarker for managing mCRC driven by EGFR signaling.

## Results

### USP21 stabilizes EGFR by deubiquitinating EGFR in colon cancer cells

**Fig. 1:**
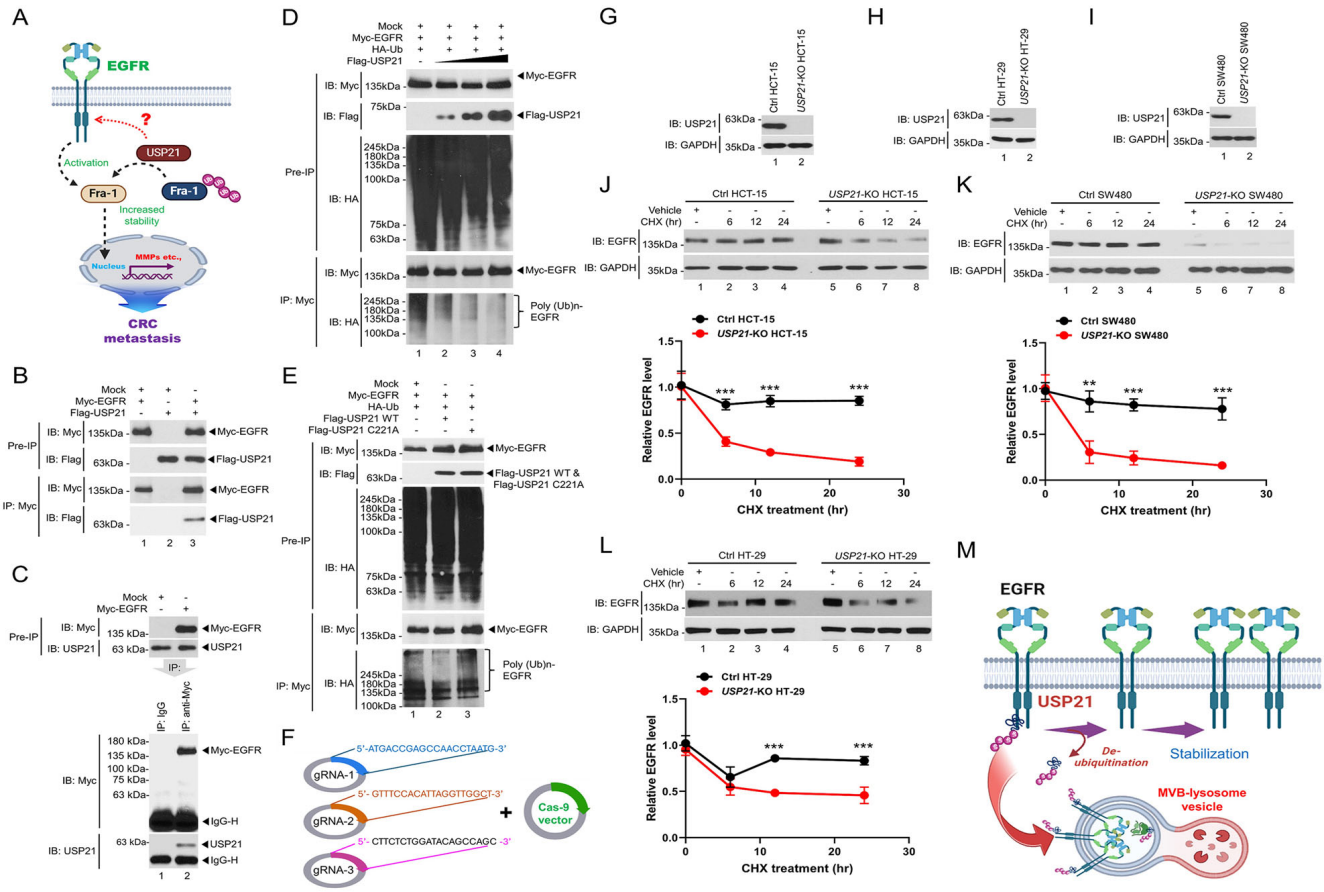
USP21 stabilizes EGFR by deubiquitinating EGFR. **A** A schematic illustration of the functional relationship between USP21 and Fra-1 or EGFR and Fra-1 in CRC metastasis. **B** Myc-EGFR, Flag-USP21, and mock vectors were transfected into HEK-293T cells, as indicated. IP assay was performed with an anti-Myc antibody **C** H1299 cells were transfected with mock as control vector or Myc-EGFR, as indicated. IP assay was performed with anti-IgG or anti-Myc antibody. **D** Myc-EGFR, HA-Ub, Flag-USP21, and mock vectors were transfected into HEK-293T cells, as indicated. IP assay was performed with an anti-Myc antibody. **E** Myc-EGFR, HA-Ub, Flag-USP21 wild type (WT), Flag-USP21 C221A mutant, and mock vectors were transfected into HEK-293T cells, as indicated. IP assay was performed with an anti-Myc antibody. **F** Three sgRNA to human USP21 were designed on the CRISPR design website (http://crispr.mit.edu/). To generate *USP21*-KO colon cancer cells, sgRNA vector and Cas9 vector were used. **G**–**I** sgRNAs and Cas9 vector were transfected into HCT-15 (**G**), HT-29 (**H**), and SW480 (**I**) human colon cancer cells. Expression levels of USP21 were analyzed by western blotting assay with an anti-USP21 antibody. **J**–**L** Ctrl HCT-15 and *USP21*-KO HCT-15 cells (**J**), Ctrl SW480 and *USP21*-KO SW480 cells (**K**), or Ctrl HT-29 and *USP21*-KO HT-29 cells (**L**) were treated with vehicle or cycloheximide (CHX, 20–30 μg/mL) for different time periods, as indicated. Western blotting assay was performed with an anti-EGFR or anti-GAPDH antibody. Error bars represent ± SD of three experiments. **p < 0.01, ***p < 0.001, two-tailed unpaired *t*-test *p* values by using Gra*p*hPad Prism 5.0. **M** A schematic model of how USP21 stabilizes EGFR.

In the context of colorectal cancer (CRC) metastasis, USP21 plays a crucial role by stabilizing Fra-1 through deubiquitination, thereby promoting the expression of metastasis-related genes like MMPs [18]. Notably, Fra-1 activation is closely linked with EGFR signaling in an MMP-dependent manner [18–21]. Despite the known connections between the USP21-Fra-1 and Fra-1-EGFR pathways, the regulatory axis of USP21 and EGFR in metastatic CRC remains largely unexplored, as illustrated in Fig. 1A. Our study began by investigating the biochemical relationship between USP21 and EGFR. We observed a direct interaction between USP21 and EGFR (Fig. 1B, lane 3), which was further validated through semi-endogenous immunoprecipitation (Fig. 1C, lane 2). Notably, USP21 was found to induce the deubiquitination of EGFR in a dose-dependent manner (Fig. 1D, lane 1 vs. lanes 2–4). To determine whether this deubiquitination activity depended on USP21’s enzymatic function, we created a catalytically inactive mutant, USP21 C221A, and compared the deubiquitination effects of the wild-type (WT) USP21 and mutant USP21. The deubiquitination of EGFR occurred in the presence of WT USP21 but not with the USP21 C221A mutant (Fig. 1E, lane 3 vs. lane 2), indicating that the deubiquitination of EGFR is likely dependent on USP21’s catalytic activity.

Next, we explored whether USP21-mediated deubiquitination of EGFR affected EGFR stability. Using CRISPR-Cas9 gene editing, we generated three distinct *USP21*-KO colon cancer cell lines: *USP21*-KO HCT-15, *USP21-*KO HT-29, and *USP21*-KO SW480 (Fig. 1F, three gRNAs for USP21 and Cas-9 vector; Fig. 1G, *USP21*-KO HCT-15; Fig. 1H, *USP21*-KO HT-29; Fig. 1I, *USP21*-KO SW480). Assessing EGFR’s half-life using a cycloheximide (CHX) chase assay in control (Ctrl) and *USP21*-KO cells revealed significantly reduced EGFR levels in *USP21*-KO cells compared to their respective control counterparts (Fig. 1J, *USP21*-KO HCT-15 vs. Ctrl HCT-15; Fig. 1K, *USP21*-KO SW480 vs. Ctrl SW480; Fig. 1L, *USP21*-KO HT-29 vs. Ctrl HT-29). These results suggest that USP21 interacts with and stabilizes EGFR, possibly by preventing its ubiquitin-mediated degradation within multivesicular body (MVB)-lysosome vesicles, thereby leading to increased EGFR expression levels (Fig. 1M).

### USP21 plays a crucial role in the progression of colon cancer and impacts survival rates in patients with metastatic CRC

**Fig. 2:**
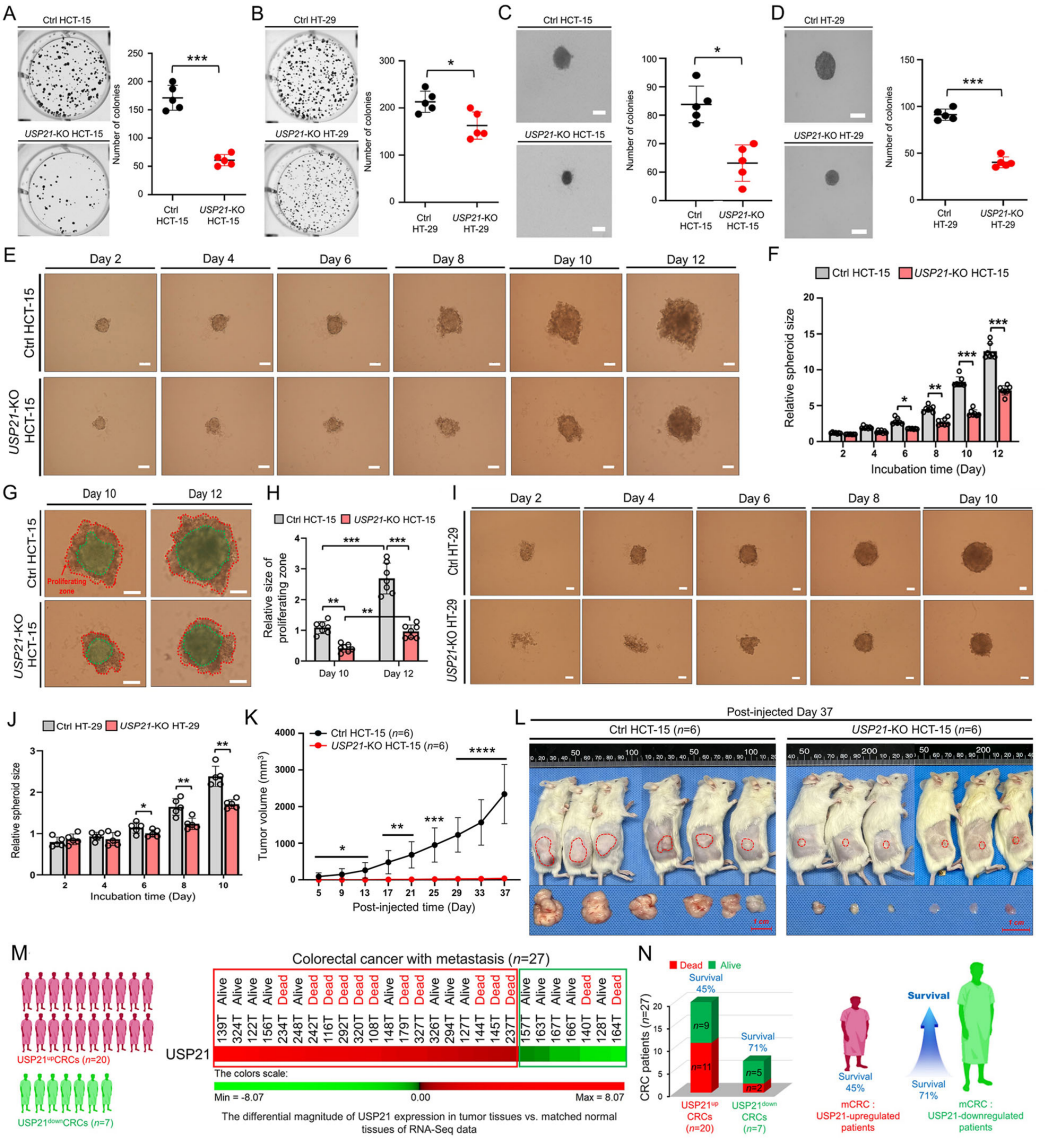
*USP21*-KO colon cancer cells exhibit attenuation of tumorigenicity both in vitro and in vivo. **A**, **B** Anchorage-dependent colony formation assay was performed with Ctrl HCT-15 and *USP21*-KO HCT-15 cells (**A**) or Ctrl HT-29 and *USP21*-KO HT-29 cells (**B**). Results are presented as mean ± SD of three independent experiments. *p < 0.05, ***p < 0.001, two-tailed unpaired *t*-test *p*-values by using GraphPad Prism 5.0. **C** and **D** Anchorage-independent colony formation assay was performed with Ctrl HCT-15 and *USP21*-KO HCT-15 cells (**C**, scale bar, 100 μm) or Ctrl HT-29 and *USP21*-KO HT-29 cells (**D**, scale bar, 100 μm). Results are presented as mean ± SD of three independent experiments. *p < 0.05, ***p < 0.001, two-tailed unpaired *t*-test *p*-values by using GraphPad Prism 5.0. **E**–**H** Ctrl HCT-15 or *USP21*-KO HCT-15 cells were seeded in 96 well plates, 3D spheroid formation assay was performed. Spheroid formation and growth were evaluated using phase-contrast microscopy (**E**, scale bar, 100 μm). The size of the spheroid was assessed using Image J Software. Error bars represent ± SD (*n* = 7) of three experiments (**F**). On day 10 and day 12 post-incubation, spheroid (blue dashed line) and non-spheroid cells (red dashed line) were analyzed (**G**, scale bar, 100 μm). Their sizes were assessed with the ImageJ Software. Non-spherical size was measured and presented as ± SD (*n* = 7) from three experiments (**H**). **p < 0.01, ***p < 0.001, two-tailed unpaired *t*-test *p*-values by using GraphPad Prism 5.0. **I** and **J** Ctrl HT-29 or *USP21*-KO HT-29 cells were seeded in 96 well plates. Plates were incubated at 37 °C for an additional 48 hours to allow the formation of 3D spheroids in culture. The spheroid was incubated for different time periods, as indicated. Spheroid formation and growth were evaluated using phase-contrast microscopy (**I**, scale bar, 100 μm). The size of the spheroid was assessed using Image J Software. Error bars represent ± SD (*n* = 5) of three experiments (**J**). *p < 0.05, **p < 0.01, two-tailed u*n*paired *t*-test *p*-values by using GraphPad Prism 5.0. **K**, **L** HCT-15 control cells (5 × 10^6^ cells per mouse, *n* = 6) or *USP21*-KO HCT-15 cells (5 × 10^6^ cells per mouse, *n* = 6) were injected subcutaneously into the back area of NSG mice. Tumor volume measurements began five days post-injection and were taken every four days until 37 days after injection, using calipers. Tumor growth curves are presented as the average tumor volume ± SEM for each group (**K**). Statistical significance is indicated as follows: *p < 0.05, **p < 0.01, ***p < 0.001, ****p < 0.0001. Photographs of the tumor-bearing NSG mice, showing tumors from mice injected with either control HCT-15 cells (*n* = 6) or *USP21*-KO HCT-15 cells (*n* = 6), were taken on day 37 post-injection (**L**), with the tumor areas marked by red dashed circles. **M**, **N** Based on RNA sequencing data of tumor tissues and adjusted matched normal tissues of mCRC patients (*n* = 27), ΔMag of USP21 expression was analyzed and listed according to the △Mag of USP21. mCRC patients were stratified into USP21^up^ mCRCs (*n* = 20) and USP21^down^ mCRCs (*n* = 7) (**M**), and the survival rate was analyzed (**N**).

Based on our biochemical findings, we investigated the role of USP21 in CRC progression through both in vitro and in vivo assays using *USP21*-KO colon cancer cells. We observed a significant reduction in transwell migration and wound healing in *USP21*-KO HCT-15, *USP21*-KO HT-29, and *USP21*-KO SW480 cells compared to their respective controls such as Ctrl HCT-15, Ctrl HT-29, and Ctrl SW480. (Fig. S1A–C, transwell migration; Fig. S2A–C, wound healing). Additionally, cell proliferation was markedly decreased in these *USP21*-KO colon cancer cells compared to the control cells (Fig. S3A: *USP21*-KO HCT-15 vs. Ctrl HCT-15; Fig. S3B: *USP21*-KO HT-29 vs. Ctrl HT-29; Fig. S3C: *USP21*-KO SW480 vs. Ctrl SW480). Furthermore, both anchorage-dependent and anchorage-independent colony formation were significantly reduced in *USP21*-KO HCT-15 and *USP21*-KO HT-29 cells compared to their respective controls (Fig. 2A, B: anchorage-dependent; Fig. 2C, D: anchorage-independent).

To assess the tumorigenic potential of USP21, we conducted in vitro 3D tumor spheroid assays and in vivo xenograft assays using NSG mice. Tumor spheroids derived from *USP21*-KO HCT-15 cells were significantly smaller than those from Ctrl HCT-15 cells (Fig. 2E, F). Additionally, the relative size of the proliferating zone increased in Ctrl HCT-15 cells after days 10 and 12, compared to *USP21*-KO HCT-15 cells, whereas a remarkable decrease could be observed in *USP21*-KO HCT-15 cells (Fig. 2G, H: *USP21*-KO HCT-15 vs. Ctrl HCT-15). Similarly, tumor spheroids grew progressively in Ctrl HT-29 cells but were notably smaller in *USP21*-KO HT-29 cells (Fig. 2I, J: *USP21*-KO HT-29 vs. Ctrl HT-29). Importantly, xenograft assays revealed a significant reduction in tumor growth and size in NSG mice implanted with *USP21*-KO HCT-15 cells compared to those implanted with Ctrl HCT-15 cells (Fig. 2K, L), strongly indicating the critical role of USP21 in the tumorigenicity of colon cancer cells.

Following these biochemical and cellular studies, we examined the correlation between USP21 expression and survival outcomes in metastatic CRC (mCRC). We analyzed a cohort of mCRC patients (*n* = 27, Table S1), comparing USP21 expression in tumor tissues versus matched normal tissues. The patients were categorized into two groups: USP21-upregulated (USP21^up^) mCRC (*n* = 20) and USP21-downregulated (USP21^down^) mCRC (*n* = 7) (Fig. 2M; Table S2). Notably, patients with higher USP21 expression had poorer survival outcomes, with survival rates of 45% in USP21^up^ mCRCs compared to 71% in USP21^down^ mCRCs (Fig. 2N). These findings suggest that USP21 expression plays a pivotal role in the tumorigenicity of colon cancer cells and is associated with poorer survival outcomes in mCRC patients.

### USP21 and EGFR expression is associated with poor survival in mCRC patients and USP21 promotes CRC progression in response to EGF

**Fig. 3:**
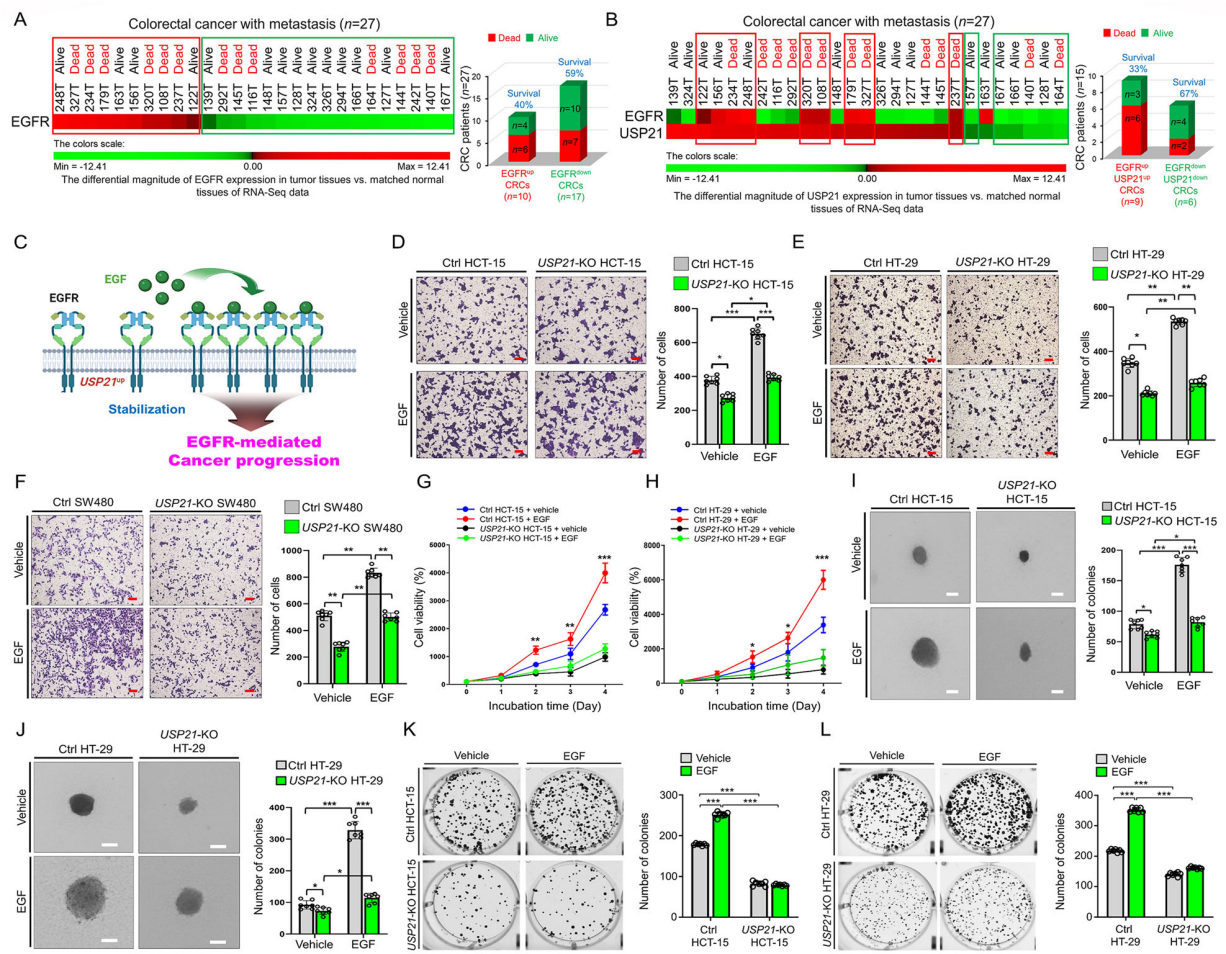
USP21 is functionally involved in colon cancer proliferation and colony formation in response to EGF. **A** Based on RNA sequencing data of tumor tissues and adjusted matched normal tissues of mCRC patients (*n* = 27), ΔMag of EGFR expression was analyzed and listed according to the ΔMag of EGFR. mCRC patients were stratified into EGFR^up^ mCRCs (*n* = 10) and EGFR^down^ mCRCs (*n* = 17), and the survival rate was analyzed. **B** Based on RNA sequencing data of tumor tissues and adjusted matched normal tissues of mCRC patients (*n* = 27), differential magnitude (ΔMag) of EGFR and USP21 expression was analyzed and listed according to the ΔMag of EGFR. mCRC patients were stratified into EGFR^up^USP21^up^ mCRCs (*n* = 9) and EGFR^down^USP21^down^ mCRCs (*n* = 6), and the survival rate was analyzed. **C** A possible model of how the up-regulated USP21 is implicated in EGFR-mediated cancer progression. **D**–**F** Transwell migration assay was performed with Ctrl HCT-15 and *USP21*-KO HCT-15 cells (**D**, scale bar, 100 μm), Ctrl HT-29 and *USP21*-KO HT-29 cells (**E**, scale bar, 100 μm), or Ctrl SW480 and *USP21*-KO SW480 cells (**F**, scale bar, 100 μm) treated with vehicle (0.01% DMSO) or EGF (20 ng/mL). Results are presented as mean ± SD of three independent experiments. *p < 0.05, **p < 0.01, ***p < 0.001, two-tailed unpaired *t* test *p*-values by using GraphPad Prism 5.0. **G**, **H** Cell proliferation assay was assay was performed with Ctrl HCT-15 and *USP21*-KO HCT-15 cells (**G**) or Ctrl HT-29 and *USP21*-KO HT-29 cells (**H**) treated with vehicle (0.01% DMSO) or EGF (20 ng/mL). Results are presented as mean ± SD of three independent experiments. *p < 0.05, **p < 0.01, ***p < 0.001, two-tailed unpaired *t*-test *p*-values by using GraphPad Prism 5.0. **I**, **J** Anchorage-independent colony formation assay was performed with Ctrl HCT-15 and *USP21*-KO HCT-15 cells (**I**, scale bar, 100 μm) or Ctrl HT-29 and *USP21*-KO HT-29 cells (**J**, scale bar, 100 μm) treated with vehicle (0.01% DMSO) or EGF (20 ng/mL). The number of colonies was counted. Results are presented as mean ± SD of three independent experiments. *p < 0.05, ***p < 0.001, two-tailed unpaired *t*-test *p*-values by using GraphPad Prism 5.0. **K**, **L** Anchorage-dependent colony formation assay was performed with Ctrl HCT-15 and *USP21*-KO HCT-15 cells (**K**) or Ctrl HT-29 and *USP21*-KO HT-29 cells (**L**) treated with vehicle (0.01% DMSO) or EGF (20 ng/mL). The number of colonies was counted. Results are presented as mean ± SD of three independent experiments. ***p < 0.001, two-tailed unpaired *t* test *p* values by using GraphPad Prism 5.0.

Given the above results, we further investigated the relationship between USP21 and EGFR expression in 27 mCRC patients. We analyzed the differential expression levels of EGFR and USP21 in CRC tumor tissues compared to matched normal tissues from these patients (Table S2). Notably, patients with EGFR-upregulated (EGFR^up^) mCRC, or a combination of EGFR^up^ and USP21-upregulated (EGFR^up^USP21^up^) mCRC, had lower survival rates than those with EGFR-downregulated (EGFR^down^) or EGFR^down^USP21^down^ mCRC (Fig. 3A: 40% in EGFR^up^ vs. 59% in EGFR^down^; Fig. 3B: 33% in EGFR^up^USP21^up^ vs. 67% in EGFR^down^USP21^down^). Specifically, survival rates for EGFR^up^USP21^up^ mCRC patients were significantly lower than those for patients with either EGFR^up^ or USP21^up^ alone (Fig. 3B vs. Figure 2N or Fig. 3A: 33% in EGFR^up^USP21^up^ vs. 45% in USP21^up^ or 40% in EGFR^up^), suggesting a potential link between EGFR and USP21 expression in influencing survival outcomes. This correlation may be associated with USP21-mediated stabilization of EGFR (Fig. 1M), potentially enhancing EGFR-driven cancer progression, as illustrated in Fig. 3C.

To confirm the functional role of USP21, we investigated its contribution to CRC progression following EGFR stimulation. We assessed the ability of *USP21*-KO cells to promote colon cancer progression upon exposure to EGF. After EGF stimulation, *USP21*-KO HCT-15, *USP21*-KO HT-29, and *USP21*-KO SW480 cells showed significantly reduced transwell migration and wound healing abilities compared to their respective control cells (Fig. 3D–F for transwell migration; Fig. S4A–F for wound healing). Similar trends were observed in cell proliferation assays with EGF-stimulated *USP21*-KO CRC cells (Fig. 3G: *USP21*-KO HCT-15 vs. Ctrl HCT-15; Fig. 3H: *USP21*-KO HT-29 vs. Ctrl HT-29; Fig. S5: *USP21*-KO SW480 vs. Ctrl SW480). Additionally, anchorage-independent and anchorage-dependent colony formation assays revealed a significant reduction in colony numbers for *USP21*-KO HCT-15, *USP21*-KO HT-29, and *USP21*-KO SW480 cells treated with either vehicle or EGF compared to control cells (Figs. 3I, J and S6A: anchorage-independent; Figs. 3K, L and S6B: anchorage-dependent). These findings underscore the role of USP21 in regulating colon cancer proliferation, migration, and colony formation in response to EGF.

**Fig. 4:**
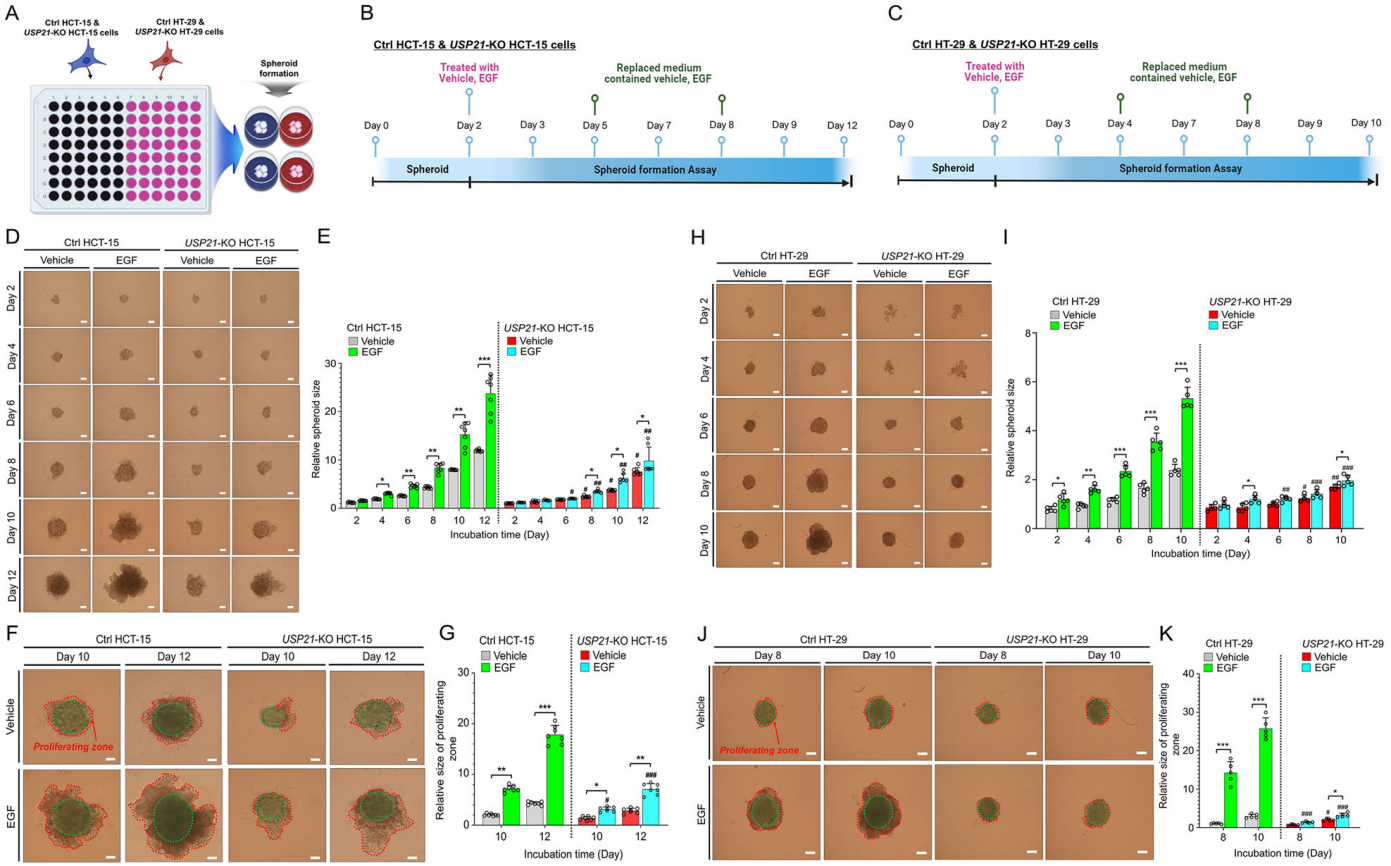
3D tumor spheroid formation is decreased in *USP21*-KO colon cancer cells in response to EGF. **A**-**C** A procedure of 3D tumor spheroid formation. Ctrl HCT-15 and *USP21*-KO HCT-15 cells (**A** and **B**) or Ctrl HT-29 and *USP21*-KO HT-29 cells (**A** and **C**) were seeded into 96-well plates at a concentration of 500 cells per well. These 96-well plates were then incubated at 37 °C for an additional 48 hours to allow the formation of 3D spheroids in culture. The spheroid was added with vehicle (0.01% DMSO) and EGF (20 ng/mL) and incubated for different time periods. The culture medium containing vehicle or EGF was exchanged with the time interval of day 3 as indicated (**B** and **C**). **D**-**G** Spheroids of Ctrl HCT-15 and *USP21*-KO HCT-15 cells were treated with vehicle or EGF and incubated for different time periods, as indicated (**D**, scale bar, 100 μm). Spheroid formation and growth were evaluated using phase-contrast microscopy. Sizes of spheroids were assessed using the Image J Software. Error bars represent ± SD (*n* = 7) of three experiments (**E**). On day 10 and day 12 post-incubation, the size of proliferating zone (red dashed line) was analyzed (**F**, scale bar, 100 μm). Their sizes were assessed with the ImageJ Software and presented as ± SD (*n* = 7) (**G**). *p < 0.05, **p < 0.01, ***p < 0.001; ^#^*p* < 0.05, ^##^*p* < 0.01, ^###^*p* < 0.001, *USP21*-KO HCT-15 versus Ctrl HCT-15, two-tailed unpaired *t*-test *p*-values by using GraphPad Prism 5.0. **H**-**K** Spheroids of Ctrl HT-29 and *USP21*-KO HT-29 cells were treated with vehicle or EGF and incubated for different time periods, as indicated (**H**, scale bar, 100 μm). Spheroid formation and growth were evaluated using phase-contrast microscopy. Sizes of spheroids were assessed using the Image J Software. Error bars represent ± SD (*n* = 5) (**I**) On day 8 and day 10 post-incubation, the size of proliferating zone (red dashed line) was analyzed (**J**, scale bar, 100 μm). Their sizes were assessed with the ImageJ Software and presented as ± SD (*n* = 5) (**K**). *p < 0.05, **p < 0.01, ***p < 0.001; ^#^*p* < 0.05, ^##^*p* < 0.01, ^###^*p* < 0.001, *USP21*-KO HT-29 versus Ctrl HT-29, two-tailed unpaired *t*-test *p*-values by using GraphPad Prism 5.0.

We further investigated the role of USP21 in EGF-induced 3D tumor spheroid formation. Ctrl HCT-15 and *USP21*-KO HCT-15 cells, along with Ctrl HT-29 and *USP21*-KO HT-29 cells, were seeded in 96-well plates. After two days of optimizing spheroid formation, the spheroids were treated with either vehicle or EGF for various durations (Fig. 4A, B: Ctrl HCT-15 and *USP21*-KO HCT-15 cells; Fig. 4A, C: Ctrl HT-29 and *USP21*-KO HT-29 cells). In Ctrl HCT-15 cells, tumor spheroids significantly increased in size following EGF treatment compared to vehicle-treated cells (Fig. 4D, E: Ctrl HCT-15 treated with EGF vs. Ctrl HCT-15 treated with vehicle). Conversely, in *USP21*-KO HCT-15 cells, the size of tumor spheroids was noticeably smaller when treated with vehicle compared to vehicle-treated Ctrl HCT-15 cells (Fig. 4D, E: *USP21*-KO HCT-15 treated with vehicle vs. Ctrl HCT-15 treated with vehicle). Importantly, EGF-treated *USP21*-KO HCT-15 cells developed significantly smaller tumor spheroids compared to EGF-treated Ctrl HCT-15 cells (Fig. 4D, E: *USP21*-KO HCT-15 treated with EGF vs. Ctrl HCT-15 treated with EGF). By days 10 and 12 post-incubation, the relative size of the proliferating zone was significantly smaller in *USP21*-KO HCT-15 cells treated with vehicle or EGF compared to Ctrl HCT-15 cells (Fig. 4F, G: *USP21*-KO HCT-15 vs. Ctrl HCT-15). Similar results were observed in 3D tumor spheroid formation assays conducted with *USP21*-KO HT-29 cells compared to Ctrl HT-29 cells (Fig. 4H–K). These findings suggest that USP21 plays a crucial role in enhancing EGF-driven tumor formation and invasion.

### BAY-805, an inhibitor of USP21, markedly attenuates the 3D tumor formation induced by EGF treatment

**Fig. 5:**
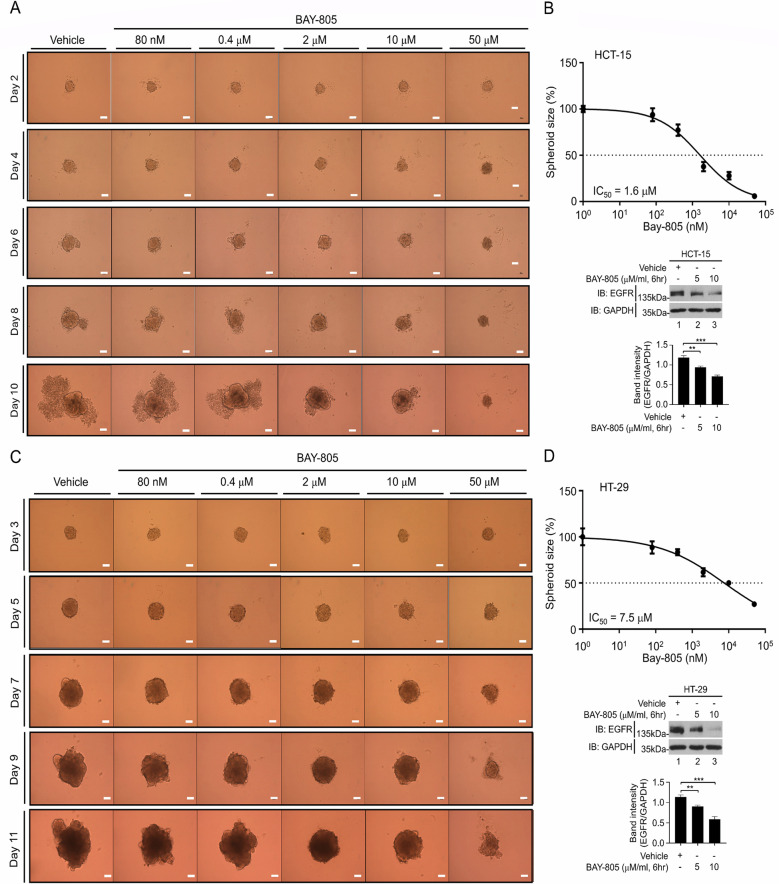
The determination of IC_50_ value of BAY-805 in wild-type (WT) HCT-15 and WT HT-29 tumor spheroids. **A**, **B** WT HCT-15 cells were seeded into 96-well plates at a concentration of 500 cells per well. These 96-well plates were then incubated at 37 °C for an additional 48 hours to allow the formation of 3D spheroids in culture. The spheroid was added with vehicle (0.01% DMSO) or different concentrations of BAY-805, as indicated. Spheroids were incubated for different time periods (**A**, scale bar, 100 μm). IC_50_ value of BAY-805 was calculated by GraphPad Prism 5.0 software (*n* = 7, spheroids). WT HCT-15 cells were treated with vehicle or different concentrations of BAY-805, as indicated (**B**, down). The levels of EGFR were evaluated by western blotting analysis with anti-EGFR antibody. The band intensity of EGFR versus GAPDH was evaluated by the ImageJ (*n* = 3). **p < 0.01, ***p < 0.001, two-tailed unpaired *t*-test *p*-values by using GraphPad Prism 5.0. **C**, **D**. WT HT-29 cells were seeded into 96-well plates at a concentration of 500 cells per well. These 96-well plates were then incubated at 37 °C for an additional 48 hours to allow the formation of 3D spheroids in culture. The spheroid was added with vehicle (0.01% DMSO) or different concentrations of BAY-805, as indicated. Spheroids were incubated for different time periods (**C**, scale bar, 100 μm). IC_50_ value of BAY-805 was calculated by GraphPad Prism 5.0 (*n* = 7, spheroids). WT HT-29 cells were treated with vehicle or different concentrations of BAY-805, as indicated (**D**, down). The levels of EGFR were evaluated by western blotting analysis with anti-EGFR antibody. The band intensity of EGFR versus GAPDH was evaluated by the ImageJ (*n* = 3). **p < 0.01, ***p < 0.001, two-tailed unpaired *t*-test *p*-values by using GraphPad Prism 5.0.

**Fig. 6:**
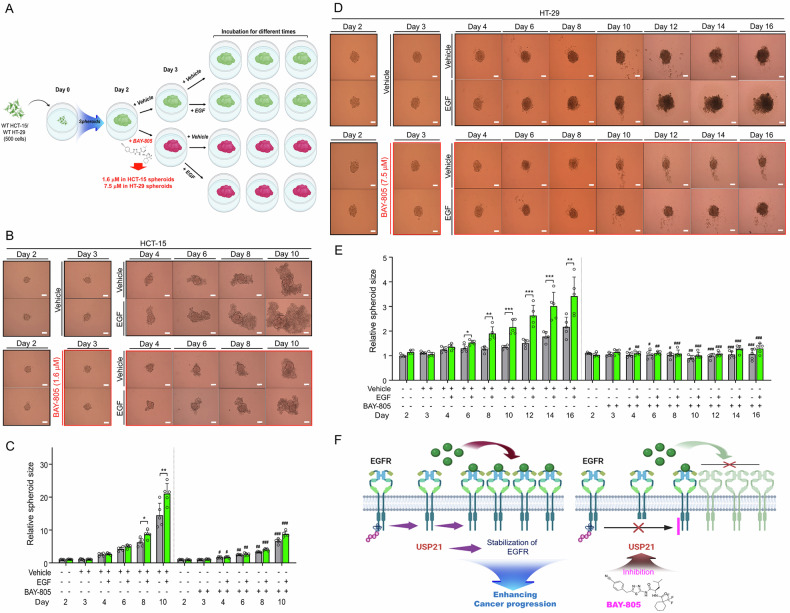
BAY-805, an USP21 inhibitor, effectively attenuates HCT-15- or HT-29- tumor spheroid formation in response to EGF stimulation. **A** A procedure for evaluating BAY-805 effects in 3D tumor spheroid formation. Wild-type (WT) HT-29 or WT HCT-15 cells were seeded into 96-well plates at a concentration of 500 cells per well. These 96-well plates were then incubated at 37 °C for an additional 48 hours to allow the formation of 3D spheroids in culture, and spheroids were treated with vehicle (0.01% DMSO) or 7.5 μM BAY-805 in HT-29 spheroids and 1.6 μM BAY-805 in HCT-15 spheroids. After 24 hr, spheroids were further treated with vehicle (0.01% DMSO) or EGF (20 ng/mL). **B**, **C** HCT-15-spheroid formation and growth were evaluated using phase-contrast microscopy (**B**, scale bar, 100 μm). Sizes of spheroids were assessed using the Image J Software. Error bars represent ± SD (*n* = 5) of three experiments (**C**). *p < 0.05, **p < 0.01; ^#^*p* < 0.05, ^##^*p* < 0.01, ^###^*p* < 0.001, HCT-15 treated without BAY-805 versus HCT-15 treated with BAY-805, two-tailed unpaired *t*-test *p*-values by using GraphPad Prism 5.0. **D** and **E** HT-29-spheroid formation and growth were evaluated using phase-contrast microscopy (**D**, scale bar, 100 μm). Sizes of spheroids were assessed using the Image J Software. Error bars represent ± SD (*n* = 5) of three experiments (**E**). *p < 0.05, **p < 0.01, ***p < 0.001; ^#^*p* < 0.05, ^##^*p* < 0.01, ^###^*p* < 0.001, HT-29 treated without BAY-805 versus HT-29 treated with BAY-805, two-tailed un*p*aired *t*-test *p*-values by using GraphPad Prism 5.0. **F** A schematic model of how USP21 inhibitor attenuates EGFR-mediated cancer progression.

Having established that USP21 contributes to tumor formation triggered by EGF stimulation, we explored the impact of inhibiting USP21 activity using the pharmacological inhibitor BAY-805 on EGF-induced tumor formation. We first determined the IC_50_ value of BAY-805 for inhibiting spheroid formation in HCT-15 and HT-29 cells (Fig. 5A, B for HCT-15; Fig. 5C, D for HT-29). The 3D tumor spheroids from these cells were treated with either a vehicle or various concentrations of BAY-805 (ranging from 80 nM to 50 μM), as shown (Fig. 5A for HCT-15; Fig. 5C for HT-29). The IC_50_ value of BAY-805 was determined to be 1.6 μM in HCT-15 spheroids and 7.5 μM in HT-29 spheroids (Fig. 5B for HCT-15; Fig. 5D for HT-29). Notably, BAY-805 treatment significantly reduced EGFR expression in both HCT-15 and HT-29 cells compared to vehicle treatment (Fig. 5B, lower panel, western blot for HCT-15; Fig. 5D, lower panel, western blot for HT-29). This suggests that inhibiting USP21 with BAY-805 leads to a reduction in EGFR expression. To evaluate the therapeutic effects of BAY-805 on EGF-induced spheroid formation, HCT-15 and HT-29 spheroids were treated with either a vehicle or EGF, in the presence or absence of BAY-805 (1.6 μM for HCT-15; 7.5 μM for HT-29) for varying durations, as shown in Fig. 6A. EGF treatment significantly increased the size of tumor spheroids in both cell lines when BAY-805 was absent, compared to vehicle treatment without BAY-805 (Fig. 6B, C: HCT-15 + EGF without BAY-805 vs. HCT-15 + vehicle without BAY-805; Fig. 6D, E: HT-29 + EGF without BAY-805 vs. HT-29 + vehicle without BAY-805). Notably, BAY-805 treatment significantly inhibited the formation of both HCT-15 and HT-29 spheroids in response to EGF, compared to those without BAY-805 (Fig. 6B, C: HCT-15 + EGF with BAY-805 vs. HCT-15 + EGF without BAY-805; Fig. 6D, E: HT-29 + EGF with BAY-805 vs. HT-29 + EGF without BAY-805). These results suggest that USP21 promotes EGF-induced cancer progression by stabilizing EGFR, and inhibiting USP21 activity can reduce EGF-induced cancer progression, as illustrated in Fig. 6F.

## Discussion

EGFR is overexpressed in 60–80% of colorectal cancers (CRCs), making anti-EGFR targeting a crucial strategy in CRC treatment [23–25]. As a multifunctional receptor, EGFR plays a vital role in various cellular processes, including cell division, differentiation, migration, and organogenesis [25, 26]. Dysregulated EGFR signaling significantly affects multiple pathways, such as PLC-gamma-1, RAS-RAF-MEK-MAPKs, phosphatidylinositol-3 kinase and Akt, Src, stress-activated protein kinases, PAK-JNKK-JNK, and signal transducers and activators of transcription [24]. Consequently, developing inhibitors targeting the EGFR signaling pathway has become a promising approach in treating various cancers, including CRC [27].

The regulation of EGFR stability and expression has garnered attention as a potential therapeutic target due to the importance of EGFR regulation. USPs, which play a role in the recycling and degradation pathway of EGFR, have emerged as targets for intervening in EGFR-induced cancer development and progression [8–10, 28–30]. Several USPs have been identified as modulators affecting EGFR stability [13–17]. In cancer medicine, there is a growing focus on pharmacologically disrupting USP activity to specifically target cancer-causing protein abnormalities, including EGFR [26, 27]. Among the USPs, USP21 is notable for interacting with multiple substrate proteins and is considered a critical oncogene in various human cancers [31–34]. Recent findings also suggest its involvement in CRC metastatic progression by stabilizing Fra-1, a protein linked to tumor formation and metastasis [18]. By increasing the expression of MMP-1 and Fra-1 target genes, USP21 influences CRC progression. Given USP21’s role in regulating the ubiquitin-mediated degradation pathway, there is a hypothesis that USP21 could regulate EGF-mediated CRC progression by modulating EGFR stability.

The findings in this study highlight the critical role of USP21 in CRC progression, particularly in mCRC. Through a series of in vitro and in vivo experiments, we elucidated the molecular mechanisms underlying USP21-mediated regulation of EGFR stability and its impact on CRC progression and patient survival. Our results demonstrate that USP21 enhances EGFR expression by stabilizing the receptor through deubiquitination, thus promoting EGFR-mediated signaling in colon cancer cells. This mechanism is crucial for maintaining EGFR levels, as shown by the reduced EGFR expression and impaired tumorigenic potential in *USP21*-KO colon cancer cells. Furthermore, we demonstrate that USP21’s enzymatic activity is essential for its ability to deubiquitinate and stabilize EGFR, underscoring its functional significance in CRC progression.

Importantly, our study reveals a significant association between USP21 expression levels and patient survival in mCRC. Patients with higher USP21 expression levels exhibited poorer survival outcomes compared to those with lower expression levels, suggesting that USP21 could serve as a potential prognostic marker for mCRC. Moreover, our data indicate a synergistic relationship between USP21 and EGFR expression in mCRC, where patients with concurrent upregulation of both proteins had the lowest survival rates. This finding underscores the clinical relevance of targeting the USP21-EGFR axis in mCRC therapy. We also provide evidence for USP21’s functional relevance in CRC progression in response to EGF stimulation. *USP21*-KO cells showed reduced proliferative and migratory capacities following EGF exposure, highlighting USP21’s role in mediating EGF-induced CRC progression. Additionally, our results show that the pharmacological inhibition of USP21 with BAY-805 significantly reduces EGF-induced tumor formation in vitro, suggesting the therapeutic potential of targeting USP21 in mCRC treatment.

**Fig. 7:**
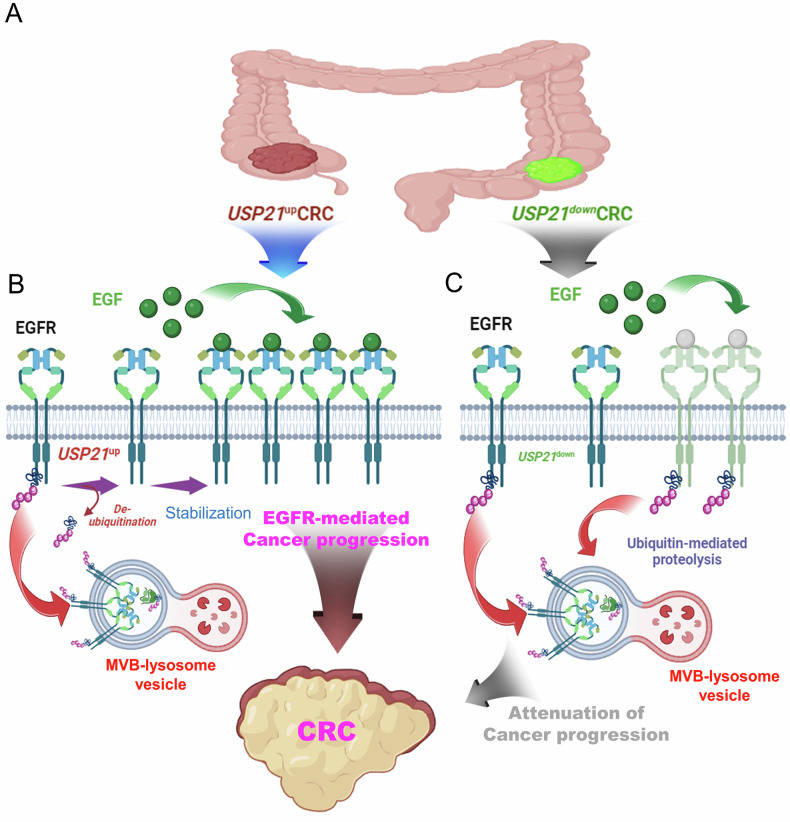
A schematic representation of the proposed model illustrating how USP21 promotes EGFR-mediated progression in CRC patients. **A** Based on USP21 expression levels in CRC tumor tissues, tumors are categorized into USP21-upregulated (USP21^up^) CRC or USP21-downregulated (USP21^down^) CRC. **B** In tumors with upregulated USP21, the enzyme inhibits the ubiquitin-dependent degradation pathway within MVB-lysosome vesicles by deubiquitinating EGFR, consequently increasing EGFR expression in CRC tumors. Subsequently, aberrant EGFR signaling initiated by EGF engagement is transmitted, thereby enhancing CRC progression. **C** Conversely, in tumors with downregulated USP21, EGFR undergoes ubiquitination and subsequent degradation within MVB-lysosome vesicles, leading to attenuated cancer progression through diminished engagement with EGF.

In the proposed model depicted in Fig. 7, we illustrate a functional association between EGFR and USP21 in CRC progression. mCRC patients with upregulated USP21 may experience more aggressive cancer progression, especially in an EGF-rich tumor microenvironment, compared to those with downregulated USP21 (Fig. 7A). In tumors with upregulated USP21, the enzyme inhibits the ubiquitin-dependent degradation pathway within MVB-lysosome vesicles by deubiquitinating EGFR, thereby increasing EGFR expression in CRCs. This aberrant EGFR signaling, initiated by EGF engagement, promotes CRC progression (Fig. 7B). Conversely, in tumors with downregulated USP21, EGFR undergoes ubiquitination and subsequent degradation within MVB-lysosome vesicles, leading to reduced cancer progression due to diminished EGF engagement (Fig. 7C).

In conclusion, our study elucidates the multifaceted role of USP21 in CRC progression and patient survival, positioning USP21 as a promising therapeutic target for mCRC. Further investigation into the therapeutic efficacy of USP21 inhibitors, such as BAY-805, in preclinical and clinical settings may provide new avenues for improving the prognosis and treatment outcomes for mCRC patients.

## Material and methods

### CRC patient specimens

Primary tumor tissues and adjusted matched normal tissues of mCRC patients (*n* = 27) were collected at Samsung Medical Center (SMC, Seoul, Korea). Licensed pathologists confirmed histologic diagnoses and estimated all formalin-fixed paraffin-embedded samples with purity ≥40% according to H&E staining. Written informed consent was obtained from all participants. All methods, including authorization for utilization of patients’ specimens, were carried out in accordance with relevant guidelines and regulations. Experiments conducted on patient samples were approved by the Institutional Review Board (IRB) of Samsung Medical Center (IRB# 2010-04-004). RNA sequencing was conducted for all samples, as previously described [35].

### Xenografted NSG mouse model

NOD/SCID/IL-2Rγ^null^ (NSG) mice were purchased from the Jackson Laboratory (Bar Harbor, ME, USA) and maintained under specific pathogen-free conditions in accordance with ethical guidelines for the care of these mice at the Bioanalysis Center Animal Facility, GenNBio Inc. (Seongnam, Korea). All experimental procedures were approved by the Institutional Animal Care and Use Committee (IACUC) of the Bioanalysis Center Animal Facility (IACUC #: 23-10-01). NSG mice (6–8 weeks old) were used to generate xenografted NSG mice. Control (Ctrl) HCT-15 (5 × 10^6^ cells per mouse, *n* = 6) or *USP21*-knockout (KO) HCT-15 cells (5 × 10^6^ cells per mouse, *n* = 6) were injected under NSG mice skin (back area) within serum-free PBS. The final injection volume was 100 μL/mouse containing a 1:1 v/v mixture of ice-chilled Matrigel (BD Biosciences, La Jolla, CA, USA), which was kept on ice until injection. Five days after cancer cell injection, tumor volume was measured with a caliper every 4 days until 37 days after injection. Tumor volume (*V*, mm^3^) was calculated using the following equation: *V* = (*a*^2^ × *b*)/2 where *a* is the width of the tumor, and *b* is the length. Tumor growth curves are presented as average tumor volume ± SEM for each group in this study. The growth of tumors in xenograft NSG mice increased remarkably between days 33 and 37 post-injection. Therefore, the experiment was finished on day 37 to adhere to animal ethics guidelines. All studies involving mice were approved by IACUC.

### Cells

HCT-15 (human colorectal cancer cell line; CCL-225, American type culture collection (ATCC), Manassas, VA, USA), SW480 (human colon cancer cell line; CCL-228, ATCC), and HT-29 (human colorectal adenocarcinoma cell line; HTB-38, ATCC) were maintained in a medium recommended by ATTC, supplemented with 10% fetal bovine serum (FBS), penicillin (100 μg/mL), and streptomycin (100 μg/mL) in a 5% CO_2_ humidified atmosphere at 37 °C. Human embryonic kidney (HEK) 293 T cells (ATCC, CRL-11268) were cultured and maintained in Dulbecco’s modified Eagle’s medium (DMEM; Welgene, LM-001-05) supplemented with 10% fetal bovine serum (FBS).

### Generation of *USP21*-knockout (KO) colon cancer cell lines with CRISPR/Cas9 two vector system

To generate *USP21-*KO colon cancer cells with CRISPR/Cas9 gene editing method, we used two vector systems, single guide RNA (sgRNA) and CRISPR-associated protein 9 (Cas9) vectors. sgRNA and Cas9 vectors were kindly provided by Dr. Daesik Kim (Sungkyunkwan University School of Medicine, Suwon, Korea). Guide RNA sequences for CRISPR/Cas9 were designed on the CRISPR design website (http://crispr.mit.edu/) provided by the Feng Zhang Lab. Insert oligonucleotides for human USP21 gRNA were 5’-ATGACCGAGCCAACCTAATG-3’ (gRNA-1)/5’- GTTTCCACATTAGGTTGGCT-3’ (gRNA-2)/5’-CTTCTCTGGATACAGCCAGC-3’ (gRNA-3). Complementary oligonucleotides to guide RNAs (gRNAs) were annealed and cloned into a sgRNA vector. The sgRNA vector expressing gRNA of USP21 and Cas9 vector expressing Cas9 were transfected into HCT-15, SW480, and HT-29 colon cancer cells using Lipofectamine 2000 (Thermo Fisher Scientific, Waltham, MA, USA) according to the manufacturer’s instructions. At two weeks after transfection, colonies were isolated and single-cell selection was performed. The expression of USP21 in *USP21*-KO colon cancer cells was analyzed by western blotting assay with an anti-USP21 antibody.

### Antibodies and reagents

Anti-Myc (sc40), anti-USP21 (sc-515911), Anti-HA (sc-7392), and anti-GAPDH (sc-47724) antibodies were purchased from Santa Cruz Biotechnology (Santa Cruz, CA, USA). Anti-Flag (F3165) antibody were purchased from Sigma-Aldrich (St. Louis, MO, USA). Anti-EGFR antibody (2232S) was purchased from Cell Signaling Technology (Danvers, MA, USA). TrueBlot® secondary antibody (18-8817-33) were purchased from Rockland Immunochemicals (Pottstown, PA, USA). Goat anti-rabbit IgG (HRP) (GTX213110-01) antibody was purchased from GeneTex Inc. (Irvine, CA, USA). Rabbit anti-mouse IgG H&L (HRP) (ab6728) antibody was purchased from Abcam (Cambridge, MA, USA). Dimethyl sulfoxide (DMSO; D4540), Phosphate-buffered saline (PBS; CBP007A), glutaraldehyde (G6257-100ml), crystal violet (C6158-50g), cycloheximide (CHX; C1988), EGF (SRP3027), and thiazolyl blue tetrazolium bromide (MTT; M5655) were purchased from Sigma-Aldrich (St. Louis, MO, USA). Lipofectamine 2000 (11668019) was purchased from Thermo Fisher Scientific (Waltham, MA, USA). BAY-805 (HY-153045) was purchased from MedChemExpress (Monmouth Junction, NJ, USA).

### Plasmid constructs

EGFR-GFP (32751) and Flag-HA-USP21 (22574) vectors were purchased from Addgene (Watertown, MA, USA). pCMV-3Tag-7 (240202) and pCMV-3Tag-6 (240200) vectors were purchased from Agilent Technologies (Santa Clara, CA, USA). Using Flag-HA-USP21 plasmid, full-length USP21 was cloned into the pCMV-3Tag-6 vector to generate a Flag-USP21 vector. Using the EGFR-GFP plasmid, full-length EGFR was cloned into the pCMV-3Tag-7 vector to generate Myc-EGFR vector. Flag-USP21 C221A mutant was generated by site-directed mutagenesis using Flag-USP21 wild-type (WT) plasmid as previously described [36].

### Western blotting assay

Western blotting and immunoprecipitation (IP) assays were performed as previously described [37–40]. Briefly, cell lysates were prepared from control (Ctrl) HCT-15, Ctrl SW480, Ctrl HT-29, *USP21*-KO HCT-15, *USP21*-KO SW480, and *USP21*-KO HT-29 cells. They were then separated by sodium dodecyl sulfate-polyacrylamide gel electrophoresis (SDS-PAGE, 8–12%) and immune-probed with an anti-USP21 or anti-GAPDH antibody. HEK-293T cells were transfected with a mock control vector, Myc-EGFR, or Flag-USP21 vector. Cells were then incubated at 37 °C for 24 h. After collecting cells, cell lysates were prepared and immunoprecipitated with an anti-Myc antibody. IP complexes were separated by sodium dodecyl sulfate-polyacrylamide gel electrophoresis (SDS-PAGE, 8–12%) and immune-probed with an anti-Myc or anti-Flag antibody.

### Ubiquitination and deubiquitination assay

HEK-293T cells were transiently transfected with mock, Myc-EGFR, HA-Ub, Flag-USP21 wild type (WT), or Flag-USP21 C221A mutant vector. Cells were then incubated at 37 °C for 24 h. After collecting cells, cell lysates were prepared and immunoprecipitated with an anti-Myc antibody. IP complexes were separated by sodium dodecyl sulfate-polyacrylamide gel electrophoresis (SDS-PAGE, 8–12%) and immune-probed with an anti-Myc, anti-Flag, or anti-HA antibody.

### Cycloheximide (CHX) chase assay

Cycloheximide (CHX) chase assay was performed to determine the half-life of EGFR following previous protocols [41]. Briefly, Control (Ctrl) HCT-15, Ctrl SW480, Ctrl HT-29, *USP21*-KO HCT-15, *USP21*-KO SW480, and *USP21*-KO HT-29 cells were treated with CHX (20-30 µg/mL; Sigma-Aldrich, St. Louis, MO, USA) for different time periods. EGFR was then detected using western blotting assay with an anti-EGFR antibody.

### Wound-healing migration assay

A wound-healing migration assay was performed following previous protocols [37, 42–45]. Briefly, Control (Ctrl) HCT-15, Ctrl SW480, Ctrl HT-29, *USP21*-KO HCT-15, *USP21*-KO SW480, and *USP21*-KO HT-29 cells were seeded into 12-well plates and cultured to reach confluence. Cell monolayers were gently scratched and washed with a culture medium. After floating cells and debris were removed, cells attached to culture plates were treated with vehicle (DMSO, 0.01% v/v concentration) or EGF (20 ng/mL) for different time periods. Cell images were captured after culturing for different time periods as indicated in each experiment.

### Transwell migration assay

Ctrl HCT-15, Ctrl SW480, Ctrl HT-29, *USP21*-KO HCT-15, *USP21*-KO SW480, and *USP21*-KO HT-29 cells were suspended in a culture medium (250 μL) and added to the upper compartment of a 24-well Transwell® chamber (8 μm pore; Corning, 3422). Ctrl HCT-15, Ctrl SW480, Ctrl HT-29, *USP21*-KO HCT-15, *USP21*-KO SW480, and *USP21*-KO HT-29 cells and culture medium (250 μL) were mixed with vehicle (DMSO, 0.1% v/v concentration) or EGF (20 ng/mL) and incubated at 37 °C for 24 h. Migratory cells would pass through polycarbonate membrane and cling to the bottom side. Non-migratory cells would stay in the upper chamber. After removing non-migratory cells, migratory cells were fixed using 2.5% glutaraldehyde (Sigma-Aldrich, G6257-100 mL) and then stained with 0.1% crystal violet (Sigma-Aldrich, C6158-50g).

### Anchorage-independent soft agar colony formation assay

Anchorage-independent soft agar colony formation assay was performed following previous protocols [37, 42–45]. Briefly, Ctrl HCT-15, Ctrl HT-29, Ctrl SW480, *USP21*-KO HCT-15, *USP21*-KO HT-29, and *USP21*-KO SW480 cells (1 × 10^4^ cells /well) mixed with 0.3% Agarose (BioShop Canada, AGA001.500) in complete medium were plated onto the bottom of a 0.5% agar layer in a 6-well plate with a complete medium. Growth medium (2 mL) with vehicle (DMSO, 0.01% v/v concentration) or EGF (20 ng/mL) was added to the top of the layer and cells were incubated at 37 °C for 28 days.

### Anchorage-dependent colony formation assay

The ability of a single cell to grow into a colony was assessed by the colony formation assay as previously described [37, 42–45]. Briefly, Ctrl HCT-15, Ctrl HT-29, Ctrl SW480, *USP21*-KO HCT-15, *USP21*-KO HT-29, and *USP21*-KO SW480 cells were harvested with trypsin-EDTA and resuspended as single cells. Cells (1 × 10^3^ cells per well) were plated into 6-well plates and treated with vehicle (DMSO, 0.01% v/v concentration) or EGF (20 ng/mL). Cells were incubated for ~12 days. Colonies were stained with 0.5% crystal violet (Sigma-Aldrich, C6158-50g) for 30 min at room temperature. The number of colonies was counted using ImageJ software.

### Three-dimensional (3D) spheroids formation assay using agarose-coated plates

3D spheroids formation assay was performed following previous protocols [46, 47]. Briefly, 1.5% agarose hydrogel was added to each well of a 96-well culture plate. The plate was incubated at room temperature (RT) for 30 min. Ctrl HCT-15, Ctrl HT-29, *USP21*-KO HCT-15, and *USP21*-KO HT-29 cells were seeded at a concentration of 500 cells per well in 100 µl of growth medium. The cells were centrifuged at 2000 to 2500 rpm for 2-5 minutes to promote cell aggregation. Plates were incubated at 37 °C for an additional 48 hours to allow the formation of 3D spheroids in culture. The spheroid was added with vehicle (DMSO, 0.01% v/v concentration) or EGF (20 ng/mL) and incubated for additional time periods. Spheroid formation and growth were evaluated using phase-contrast microscopy. Sizes of spheroids and non-spherical cells were assessed using ImageJ Software (National Institutes of Health, Bethesda, MD, USA). For the determination of IC_50_ value of BAY-805 in wild-type HCT-15 or HT-29 spheroids, WT HCT-15 or WT HT-29 cells were seeded into 96-well plates at a concentration of 500 cells per well. These 96-well plates were then incubated at 37 °C for an additional 48 hours to allow the formation of 3D spheroids in culture. The spheroid was added with vehicle (0.01% DMSO) or different concentrations of BAY-805. Spheroids were incubated for different time periods. IC_50_ value of BAY-805 was calculated by GraphPad Prism 8 software. To evaluate the inhibitory effect of BAY-805 on HCT-15 or HT-29 spheroids induced by EGF, WT HT-29 or WT HCT-15 cells were seeded into 96-well plates at a concentration of 500 cells per well. These 96-well plates were then incubated at 37 °C for an additional 48 hours to allow the formation of 3D spheroids in culture, and spheroids were treated with vehicle (0.01% DMSO) or 7.5 μM BAY-805 in HT-29 spheroids and 1.6 μM BAY-805 in HCT-15 spheroids. After 24 hr, spheroids were further treated with vehicle (0.01% DMSO) or EGF (20 ng/mL). Tumor spheroid formation and growth were evaluated using phase-contrast microscopy. Sizes of spheroids were assessed using the Image J Software.

### MTT assay

Ctrl HCT-15, Ctrl SW480, Ctrl HT-29, *USP21*-KO HCT-15, *USP21*-KO SW480, and *USP21*-KO HT-29 cells were seeded into 96-well culture plates at a density of 1 × 10^3^ cells/well, treated with vehicle (DMSO, 0.01% v/v concentration) or EGF (2.5 ng/mL), and grown in a culture medium supplemented with 10% FBS for different time periods. Cell viability was measured using an MTT reagent (Sigma-Aldrich, M5655) dissolved in PBS (1 mg/mL). On the day when measurements were taken, the medium was carefully replaced with fresh RPMI + 10% FBS, added with diluted MTT (1:10, 10% MTT), and incubated at 37 °C for 3 h. After removing the incubation medium, formazan crystals were dissolved in a 100 μL solution of DMSO. MTT reduction was quantified by measuring light absorbance at 595 nm using a Bio-Rad Model 680 microplate reader (Bio-Rad, CA, USA). Each test was repeated at least four times in quadruplicate.

### RNA sequencing

Primary tumor tissues and adjusted matched normal tissues were obtained from CRC patients (*n* = 27) and RNA sequencing was done using the Illumina TruSeq RNA Sample Preparation Kit v2, as described in the previous report [35]. The experiments conducted on patient samples were approved by the institutional review board of Samsung Medical Center (IRB# 2010-04-004). Written informed consents were obtained from all participating patients. All experiments and analysis procedures were performed in accordance with the relevant guidelines and regulations.

### Statistical analysis

All data are expressed as mean ± SD (standard deviation) or mean ± SEM. Statistical significance was determined by Student’s t-test using GraphPad Prism 5.0 (GraphPad Software, San Diego, CA, USA). *P*-values are marked by asterisks (*P < 0.05; **P < 0.01; ***P < 0.001 and ****P < 0.0001).

## Supplementary information

Supplementary Information

Supplementary Table S1

Supplementary Table S2

Original data file

## Data Availability

The data that support the findings of this study are available from the corresponding author upon reasonable request.
