# Supplementary material for: USP21-EGFR signaling axis is functionally implicated in metastatic colorectal cancer
Source: Cell Death Discov. 2024 Dec 18;10:492. doi: 10.1038/s41420-024-02255-1 (PMC11655878; doi:10.1038/s41420-024-02255-1)
Supplement: Supplementary file 1 — Supplementary Information [file 41420_2024_2255_MOESM1_ESM.docx]

**Supplement Figure Legends**

**
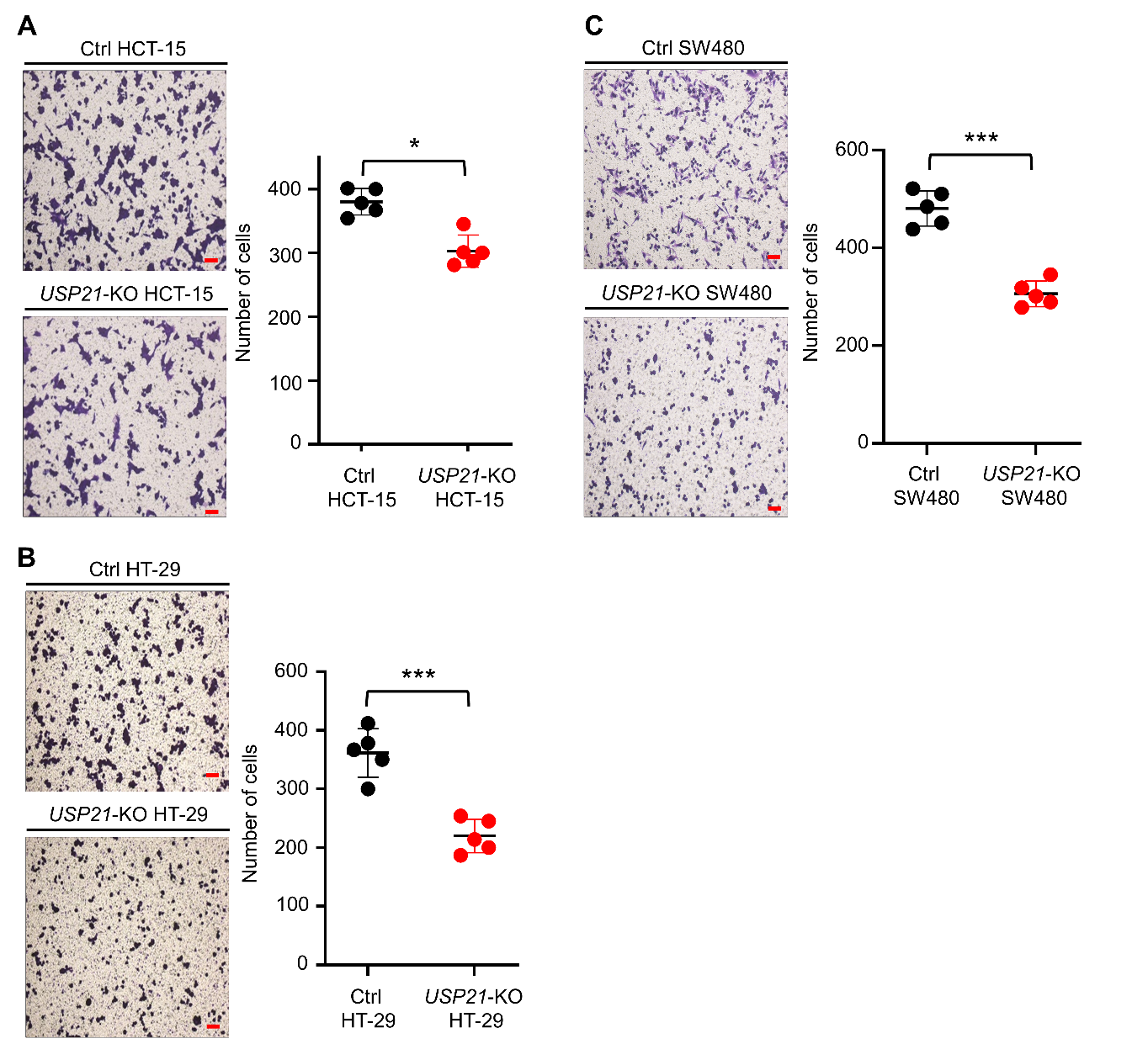
**

**Supplementary Fig. S1. A-C** Transwell migration assay was performed with Ctrl HCT-15 and *USP21*-KO HCT-15 cells (**A,** scale bar, 100 μm), Ctrl HT-29 and *USP21*-KO HT-29 cells (**B,** scale bar, 100 μm), or Ctrl SW480 and *USP21*-KO SW480 cells (**C,** scale bar, 100 μm). Results are presented as mean ± SD of three independent experiments. **p*<0.05, ****p* < 0.001, two-tailed unpaired *t*-test *p*-values by using GraphPad Prism 5.0.

**
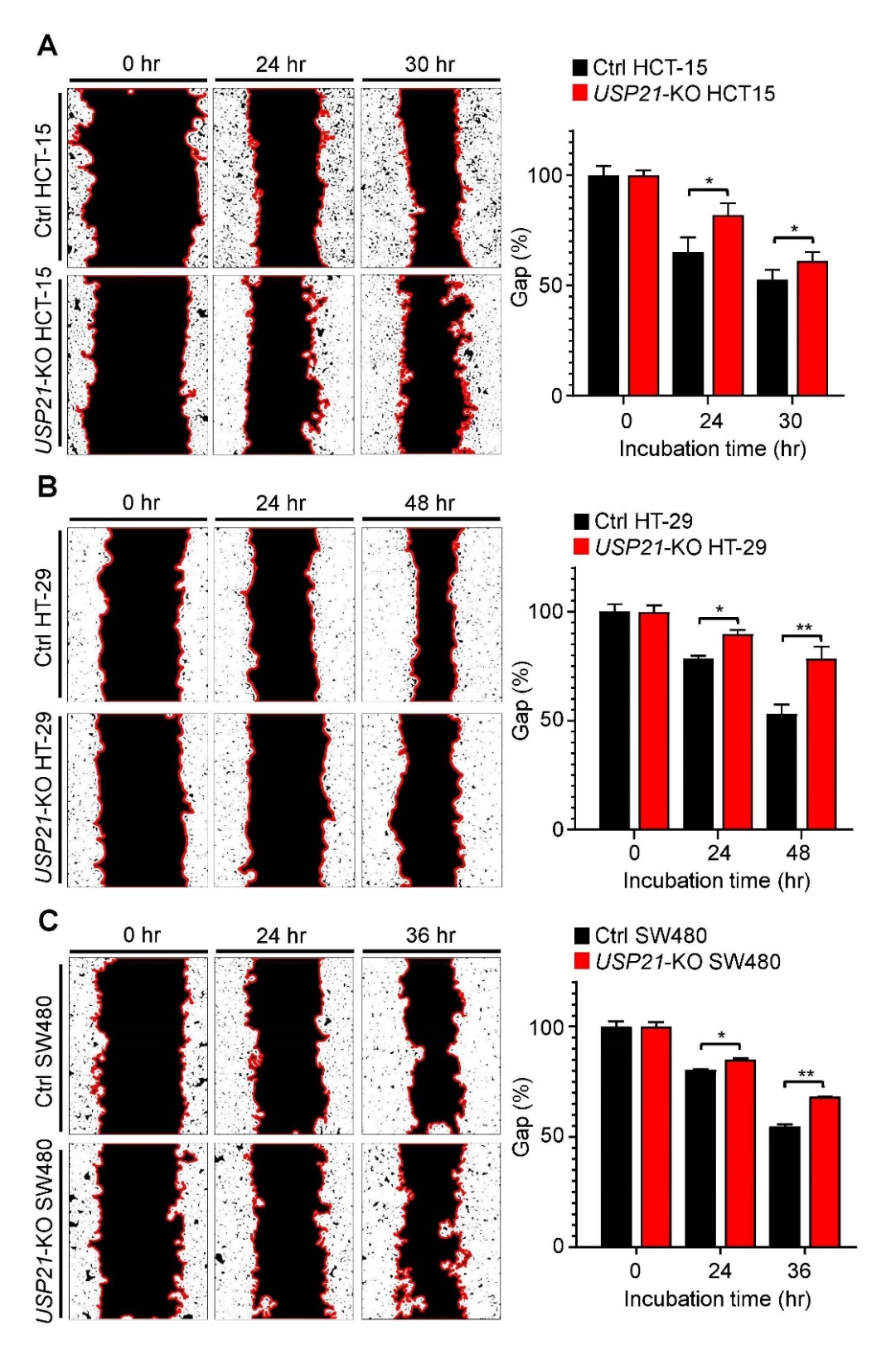
**

**Supplementary Fig. S2. A-C** Wound healing assay was performed with Ctrl HCT-15 and *USP21*-KO HCT-15 cells (**A**), Ctrl HT-29 and *USP21*-KO HT-29 cells (**B**), or Ctrl SW480 and *USP21*-KO SW480 cells (**C**). Results are presented as mean ± SD of three independent experiments. **p*<0.05, ***p* < 0.01, two-tailed unpaired *t*-test *p*-values by using GraphPad Prism 5.0.


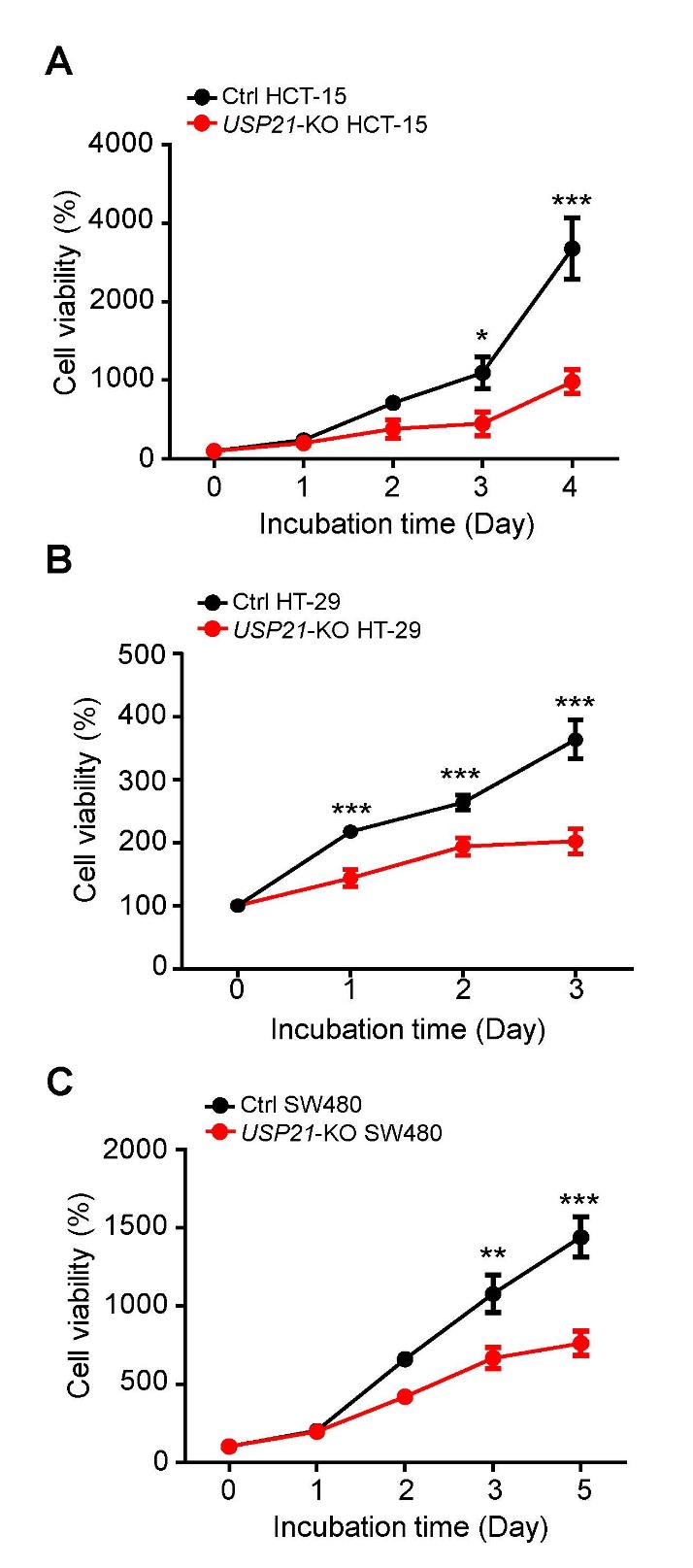


**Supplementary Fig. S3. A-C** Cell proliferation assay was performed with Ctrl HCT-15 and *USP21*-KO HCT-15 cells (**A**), Ctrl HT-29 and *USP21*-KO HT-29 cells (**B**), or Ctrl SW480 and *USP21*-KO SW480 cells (**C**). Results are presented as mean ± SD of three independent experiments. **p*<0.05, ***p* < 0.01, ****p* < 0.001, two-tailed unpaired *t*-test *p*-values by using GraphPad Prism 5.0.


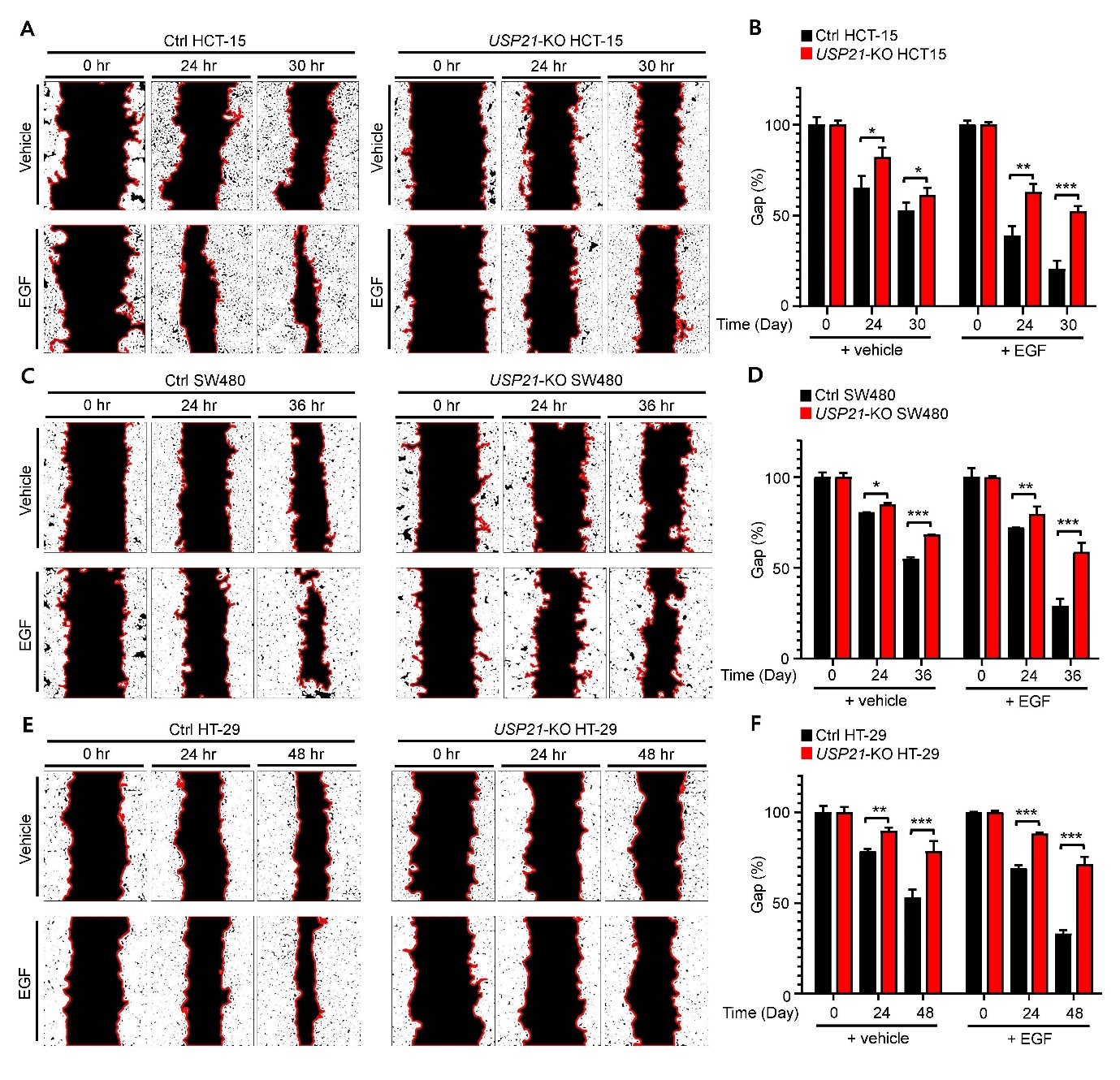


**Supplementary Fig. S4. A-F** Wound healing assay was performed with Ctrl HCT-15 and *USP21*-KO HCT-15 cells (**A** and **B**), Ctrl SW480 and *USP21*-KO SW480 cells (**C** and **D**), or Ctrl HT-29 and *USP21*-KO HT-29 cells (**E** and **F**) treated with vehicle (0.01% DMSO) or EGF (20 ng/mL). Results are presented as mean ± SD of three independent experiments. **p*<0.05, ***p* < 0.01, ****p* < 0.001, two-tailed unpaired *t*-test *p*-values by using GraphPad Prism 5.0.


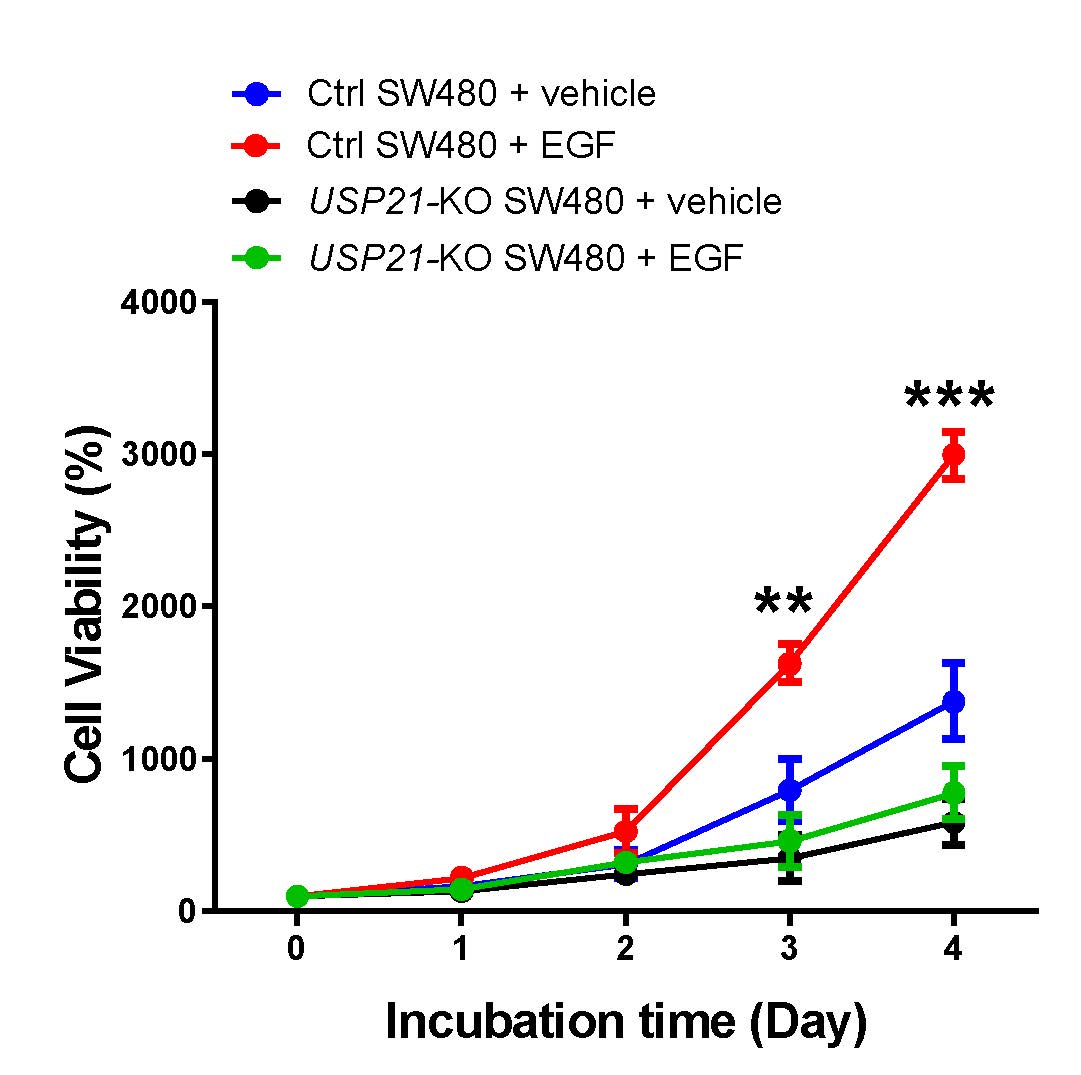


**Supplementary Fig. S5.** Cell proliferation assay was performed with Ctrl SW480 and *USP21*-KO SW480 cells treated with vehicle (0.01% DMSO) or EGF (20 ng/mL). Results are presented as mean ± SD of three independent experiments. ***p* < 0.01, ****p* < 0.001, two-tailed unpaired *t*-test *p*-values by using GraphPad Prism 5.0.


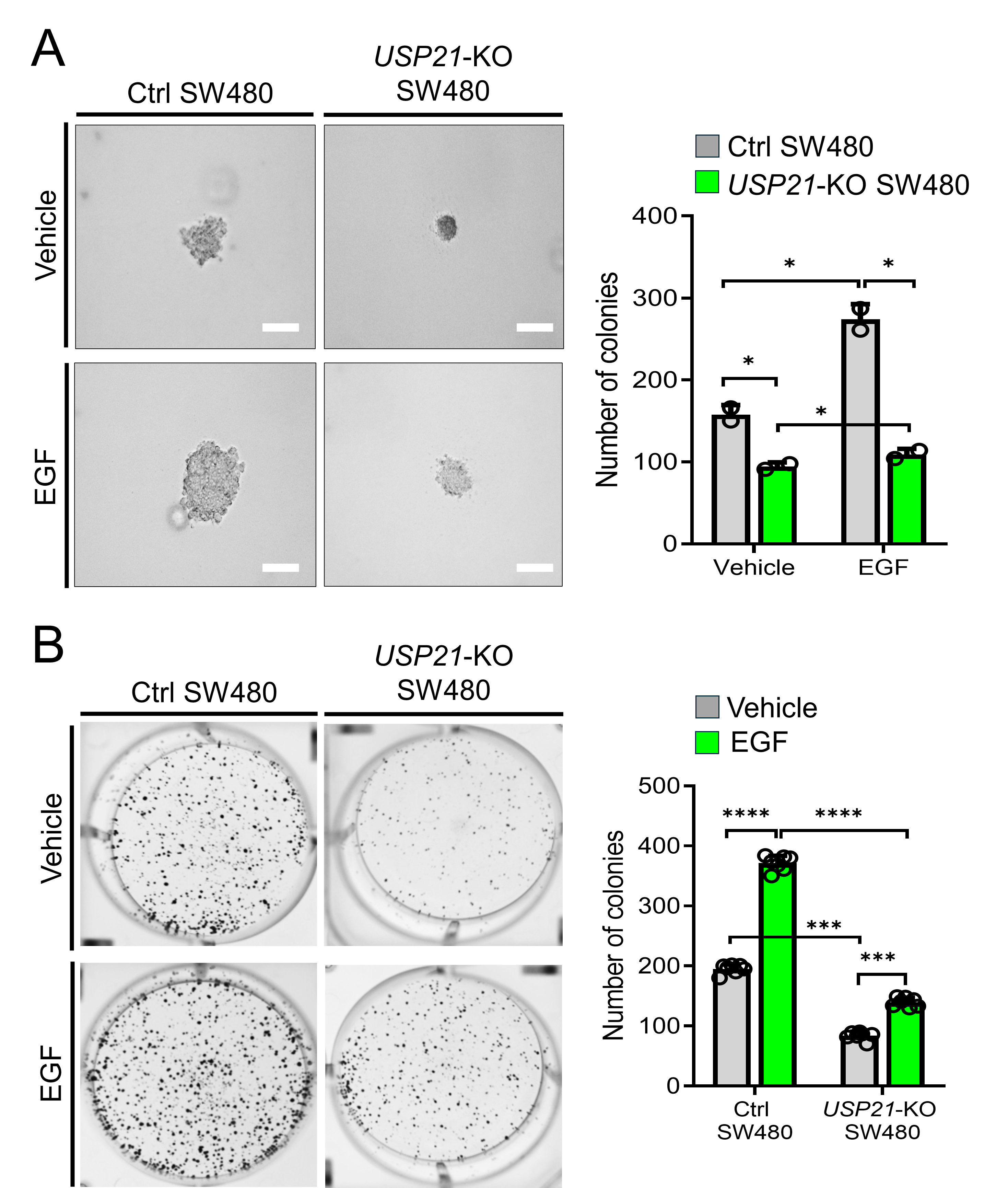


**Supplementary Fig. S6. A** and **B** Anchorage-independent (A) and -dependent (B) colony formation assay was performed with Ctrl SW480 and *USP21*-KO SW480 cells. For anchorage-independent colony formation assay, Ctrl SW480 and *USP21*-KO SW480 cells were treated with vehicle or EGF (20 ng/mL). Results are presented as mean ± SD (**A**, *n*=2). Scale bar = 100 μm. **p* < 0.05, two-tailed unpaired *t*-test *p*-values by using GraphPad Prism 5.0. For anchorage-dependent colony formation assay, Ctrl SW480 and *USP21*-KO SW480 cells were treated with vehicle or EGF (10 ng/mL). Results are presented as mean ± SD (**B**, *n*=7). ****p* < 0.001, *****p* < 0.0001, two-tailed unpaired *t*-test *p*-values by using GraphPad Prism 5.0.

**Supplement Table information**

**Supplementary Table S1.** Clinical characteristics of CRC patients (*n*=27).

**Supplementary Table S2.** Clinical characteristics of CRC patients (*n*=27) and differential expression magnitude (△Mag) of USP21 and EGFR in tumor tissues (*n*=27) vs. matched normal tissues (*n*=27).
