# Supplementary material for: USP21-EGFR signaling axis is functionally implicated in metastatic colorectal cancer
Source: Cell Death Discov. 2024 Dec 18;10:492. doi: 10.1038/s41420-024-02255-1 (PMC11655878; doi:10.1038/s41420-024-02255-1)
Supplement: Supplementary file 2 — Supplementary Table S1 [file 41420_2024_2255_MOESM2_ESM.pdf]

Supplementary Table S1. Clinical characteristics of CRC patients (*n* = 27)

Patient ID_REF	Sample_type	Age	Sex	OP_date	OP_name	Weight_kg	Height_cm	Location_ of_tumor	Metastasis	Type_of_cancer	Cell_type	Status_disease	Recur_date	Status_patient	Final_status
145T_P	Colon with Metastasis (CWM)	74	F	2011-07-01	Left hemicolectomy	54.4	142.5	descending colon	Yes	Sporadic	Adenocarcinoma, WD	Recur	2013-03-11	Dead	Death of cancer
148T_P	Colon with Metastasis (CWM)	39	F	2011-09-07	Anterior resection	79.55	161.6	sigmoid colon	Yes	Sporadic	Adenocarcinoma, MD	NETR	N/A	Alive	Alive without disease
156T_P	Colon with Metastasis (CWM)	62	F	2012-02-02	Anterior resection	45	150	sigmoid colon	Yes	Sporadic	Adenocarcinoma, MD	NETR	N/A	Alive	Alive without disease
157T_P	Colon with Metastasis (CWM)	58	M	2012-02-21	Anterior resection	76.9	173.4	sigmoid colon	Yes	Sporadic	Adenocarcinoma, PD	NETR	N/A	Alive	Alive without disease
163T_P	Colon with Metastasis (CWM)	72	M	2012-05-08	Right hemicolectomy	63.4	153	ascending colon	Yes	Sporadic	Mucinous carcinoma	Recur	2012-11-19	Alive	Alive with disease
164T_P	Colon with Metastasis (CWM)	59	F	2012-05-08	Low anterior resection	54.9	149	sigmoid colon	Yes	Sporadic	Adenocarcinoma, MD	Recur	2012-10-25	Dead	Death of cancer
166T_P	Colon with Metastasis (CWM)	59	F	2012-06-19	Anterior resection	66.6	166	sigmoid colon	Yes	Sporadic	Adenocarcinoma, MD	NETR	N/A	Alive	Alive without disease
167T_P	Colon with Metastasis (CWM)	49	F	2012-06-19	Low anterior resection	56.1	149.5	sigmoid colon	Yes	Sporadic	Adenocarcinoma, MD	NETR	N/A	Alive	Alive without disease
179T_P	Colon with Metastasis (CWM)	64	M	2012-08-06	Anterior resection	61.55	164.4	sigmoid colon	Yes	Sporadic	Adenocarcinoma, MD	Recur	2012-11-18	Dead	Death of cancer
108T_P	Colon with Metastasis (CWM)	58	M	2010-09-07	Anterior resection	70.9	168.5	sigmoid colon	Yes	Sporadic	Adenocarcinoma, MD	Recur	2011-03-21	Dead	Death of cancer
234T_P	Colon with Metastasis (CWM)	59	F	2012-10-11	Low anterior resection	56	148.3	rectum	Yes	Sporadic	Adenocarcinoma, MD	Recur	2013-11-28	Dead	Death of cancer
237T_P	Colon with Metastasis (CWM)	60	F	2012-10-26	Left hemicolectomy	62.2	163.5	splenic flexure colon	Yes	Sporadic	Adenocarcinoma, PD	Recur	2013-05-29	Dead	Death of cancer
242T_P	Colon with Metastasis (CWM)	70	M	2012-12-04	Left hemicolectomy	60.7	167.6	descending colon	Yes	Sporadic	Adenocarcinoma, MD	Recur	2013-12-17	Dead	Death of cancer
248T_P	Colon with Metastasis (CWM)	59	M	2012-12-10	Low anterior resection	56.7	174.3	rectum	Yes	Sporadic	Adenocarcinoma, WD	Recur	2014-02-03	Alive	Alive with disease
292T_P	Colon with Metastasis (CWM)	64	F	2013-02-08	Low anterior resection	45	148.3	rectosigmoid junction colon	Yes	Sporadic	Adenocarcinoma, MD	Residual tumor	N/A	Dead	Death of cancer
294T_P	Colon with Metastasis (CWM)	55	M	2013-02-12	Anterior resection	66.3	170.7	sigmoid colon	Yes	Sporadic	Adenocarcinoma, MD	Recur	2013-06-03	Alive	Alive with disease
320T_P	Colon with Metastasis (CWM)	54	M	2013-03-22	Low anterior resection	48.8	170	rectosigmoid junction colon	Yes	Sporadic	Adenocarcinoma, MD	Recur	2013-12-17	Dead	Death of cancer
324T_P	Colon with Metastasis (CWM)	63	M	2013-04-16	Anterior resection	50.1	165.2	sigmoid colon	Yes	Sporadic	Adenocarcinoma, MD	NETR	N/A	Alive	Alive without disease
116T_P	Colon with Metastasis (CWM)	56	M	2010-10-21	Anterior resection	65.9	170.6	sigmoid colon	Yes	Sporadic	Adenocarcinoma, MD	Recur	2010-12-22	Dead	Death of cancer
326T_P	Colon with Metastasis (CWM)	75	M	2013-04-18	Extended Right hemicolectomy	71	170	cecum	Yes	Sporadic	Adenocarcinoma, MD	NETR	N/A	Alive	Alive without disease
327T_P	Colon with Metastasis (CWM)	72	M	2013-04-19	Abdominoperineal resection	62.9	164.3	rectum	Yes	Sporadic	Adenocarcinoma, MD	NETR	N/A	Dead	Death of unknown causes
122T_P	Colon with Metastasis (CWM)	51	F	2010-12-02	Low anterior resection	65.3	155	rectum	Yes	Sporadic	Adenocarcinoma, MD	Recur	2011-09-28	Alive	Alive with disease
127T_P	Colon with Metastasis (CWM)	51	M	2011-01-06	Left hemicolectomy	72.1	171.3	descending colon	Yes	Sporadic	Adenocarcinoma, MD	NETR	N/A	Alive	Alive without disease
128T_P	Colon with Metastasis (CWM)	57	F	2011-01-10	Anterior resection	60.8	149.4	sigmoid colon	Yes	Sporadic	Adenocarcinoma, MD	NETR	N/A	Alive	Alive without disease
139T_P	Colon with Metastasis (CWM)	68	M	2011-05-19	Low anterior resection	56.6	168.8	rectum	Yes	Sporadic	Adenocarcinoma, WD	NETR	N/A	Alive	Alive without disease
140T_P	Colon with Metastasis (CWM)	61	F	2011-05-23	Anterior resection	61.8	145.8	sigmoid colon	Yes	Sporadic	Adenocarcinoma, MD	Recur	2012-10-30	Dead	Death of cancer
144T_P	Colon with Metastasis (CWM)	41	F	2011-06-27	Anterior resection	68.65	161.6	rectosigmoid junction colon	Yes	Sporadic	Adenocarcinoma, MD	Recur	2012-07-21	Dead	Death of cancer

Red, dead patients; green, alive patients
