# Supplementary material for: USP21-EGFR signaling axis is functionally implicated in metastatic colorectal cancer
Source: Cell Death Discov. 2024 Dec 18;10:492. doi: 10.1038/s41420-024-02255-1 (PMC11655878; doi:10.1038/s41420-024-02255-1)
Supplement: Supplementary file 3 — Supplementary Table S2 [file 41420_2024_2255_MOESM3_ESM.pdf]

Supplementary Table S2. Clinical characteristics of CRC patients (*n* =27) and differential expression magnitude (△Mag) of USP21 and EGFR in tumor tissues (*n* =27) vs. matched normal tissues (*n* =27)

Patient ID	Cancer types	Age	Sex	Patient status (Dead or Alive)	RNA Seq RPKM values in tumor tissues		RNA Seq RPKM values in matched normal tissues		Differential expression magnitude (△Mag)	
					USP21	EGFR	USP21	EGFR	USP21	EGFR
139T	CWM*	68	M	Alive	15.57529742	4.262934876	7.508325669	4.366523743	**8.06697175**	-0.103588867
324T	CWM*	63	M	Alive	12.06771899	4.988001957	7.033474017	8.409429218	**5.034244971**	-3.42142726
122T	CWM*	51	F	Alive	7.39297872	3.207919406	3.073188054	3.010804964	**4.319790665**	0.197114443
156T	CWM*	62	F	Alive	9.53996353	7.646981256	6.484234462	5.152124575	**3.055729067**	2.494856681
234T	CWM*	59	F	**Dead**	11.92290494	8.374720214	9.00103554	3.869582351	**2.921869396**	4.505137863
248T	CWM*	59	M	Alive	9.549704326	16.61638563	6.698912952	4.207499511	**2.850791374**	12.40888611
242T	CWM*	70	M	**Dead**	9.834335001	12.13458935	7.34049894	18.22134057	**2.493836061**	-6.086751216
116T	CWM*	56	M	**Dead**	11.18970821	6.571017917	8.908113481	8.306545183	**2.281594731**	-1.735527265
292T	CWM*	64	F	**Dead**	9.010575426	7.266000971	6.758294023	8.059880595	**2.252281403**	-0.793879623
320T	CWM*	54	M	**Dead**	12.41952637	11.43155691	10.19693231	10.29919066	**2.222594057**	1.132366248
108T	CWM*	58	M	**Dead**	9.965594599	8.016671229	7.86049006	6.931249013	**2.10510454**	1.085422216
148T	CWM*	39	F	Alive	10.30513619	4.99977339	8.779111485	7.003592464	**1.5260247**	-2.003819074
179T	CWM*	64	M	**Dead**	10.80134419	10.60559666	9.42403932	6.696272515	**1.377304873**	3.909324145
327T	CWM*	72	M	**Dead**	8.39610876	15.37447045	7.031465663	9.971284847	**1.364643097**	5.403185605
326T	CWM*	75	M	Alive	10.28413906	3.344837085	9.239370844	6.810289623	**1.044768213**	-3.465452539
294T	CWM*	55	M	Alive	8.307281619	6.666009521	7.338537074	10.30089766	**0.968744545**	-3.634888142
127T	CWM*	51	M	Alive	10.82601547	6.224300199	10.02682608	11.27244016	**0.799189399**	-5.048139959
144T	CWM*	41	F	**Dead**	9.229919961	6.777166452	8.524516406	11.90717128	**0.705403555**	-5.130004833
145T	CWM*	74	F	**Dead**	7.28931376	9.829578986	6.655989187	10.78289616	**0.633324573**	-0.953317173
237T	CWM*	60	F	**Dead**	6.404606619	10.70151878	5.789256638	10.05740621	**0.615349981**	0.644112569
157T	CWM*	58	M	Alive	10.53852793	7.511497654	10.73468642	10.36107846	**-0.196158488**	-2.849580804
163T	CWM*	72	M	Alive	7.439711311	13.4550057	7.717943798	10.3210201	**-0.278232487**	3.133985601
167T	CWM*	49	F	Alive	8.076912251	5.631957473	8.769586884	14.91164718	**-0.692674633**	-9.27968971
166T	CWM*	59	F	Alive	8.718145102	5.259197885	9.412852146	9.354397794	**-0.694707044**	-4.09519991
140T	CWM*	61	F	**Dead**	8.584818294	6.968102076	10.35227889	15.39759465	**-1.7674606**	-8.429492569
128T	CWM*	57	F	Alive	7.484649471	7.486645977	9.316422428	10.60041654	**-1.831772956**	-3.113770567
164T	CWM*	59	F	**Dead**	6.841407857	4.968287506	9.371641978	9.167425907	**-2.530234122**	-4.199138401

* CWM, Colon with Metastasis; alive patients indicated as blue, dead patients indicated as pink; red, up-regulated expression; green, down-regulated expression
