# Supplementary material for: USP21-EGFR signaling axis is functionally implicated in metastatic colorectal cancer
Source: Cell Death Discov. 2024 Dec 18;10:492. doi: 10.1038/s41420-024-02255-1 (PMC11655878; doi:10.1038/s41420-024-02255-1)

Figure 1B

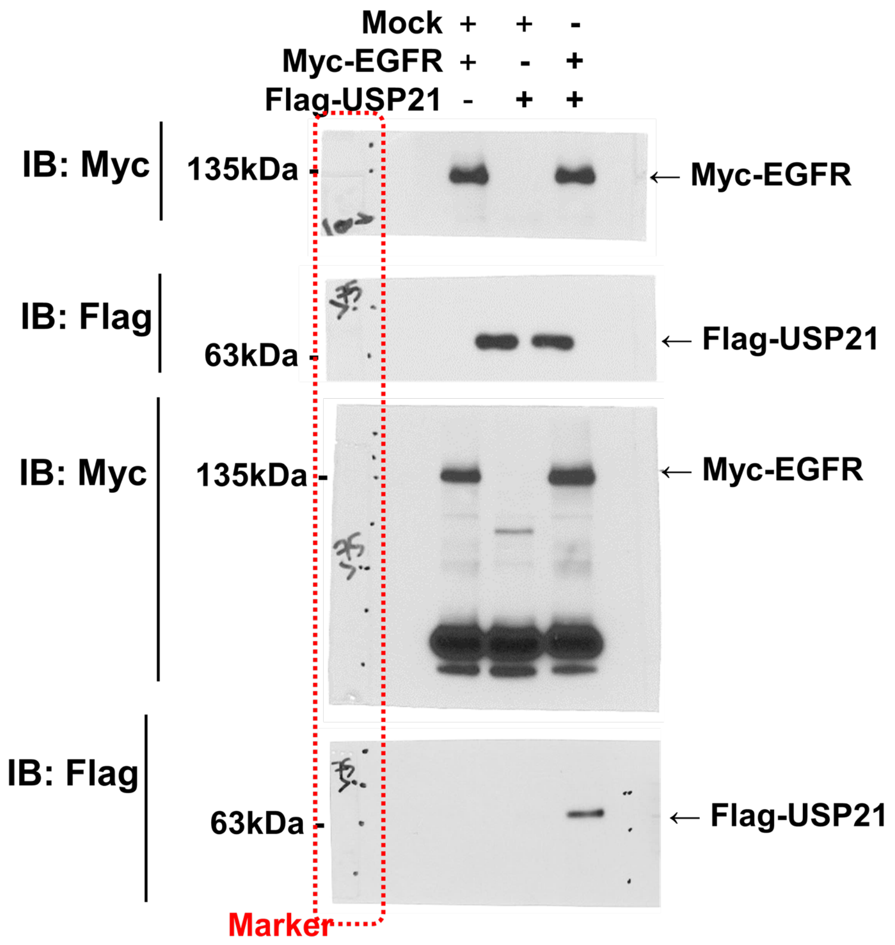

Figure 1C

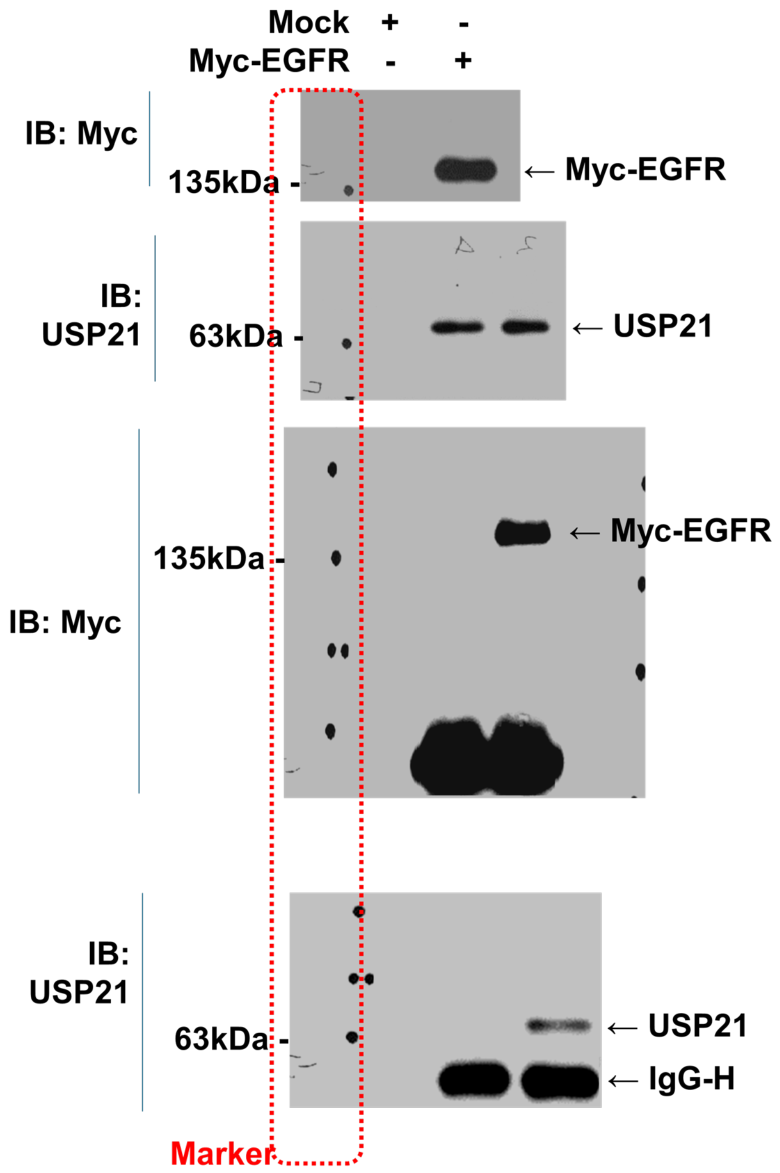

Figure 1D

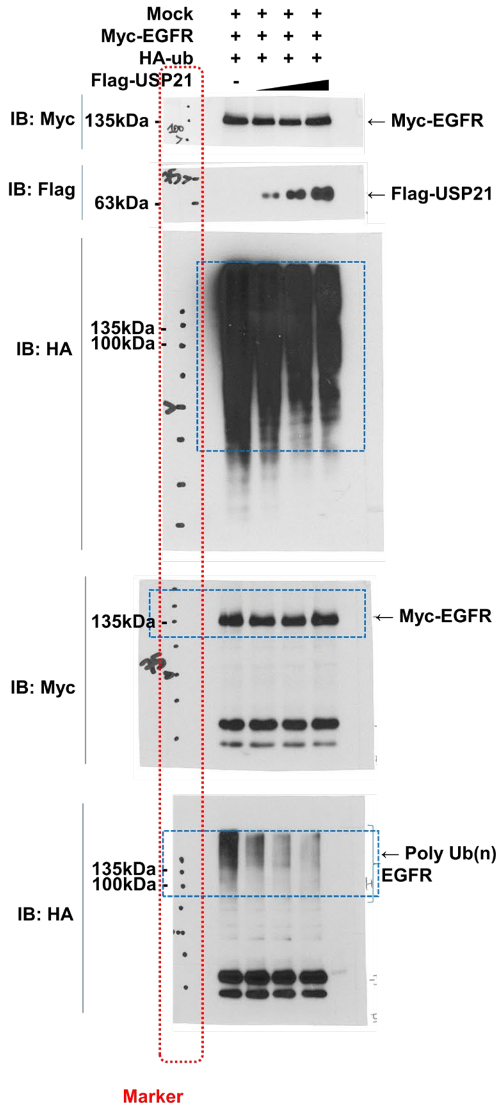

Figure 1E

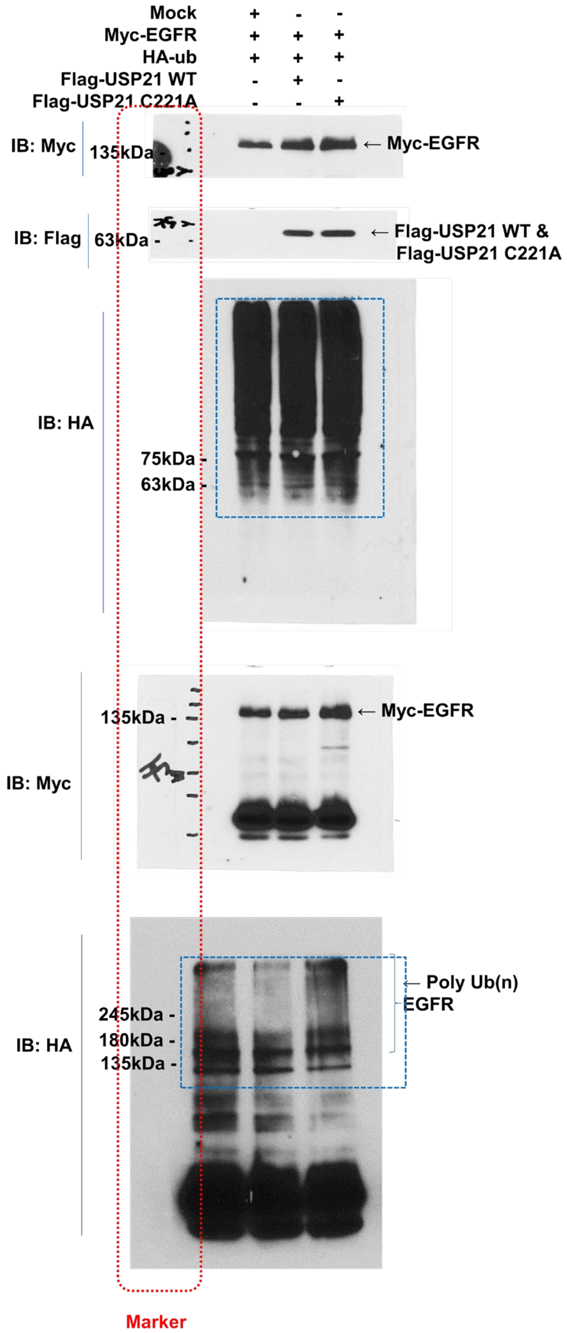

Figure 1G

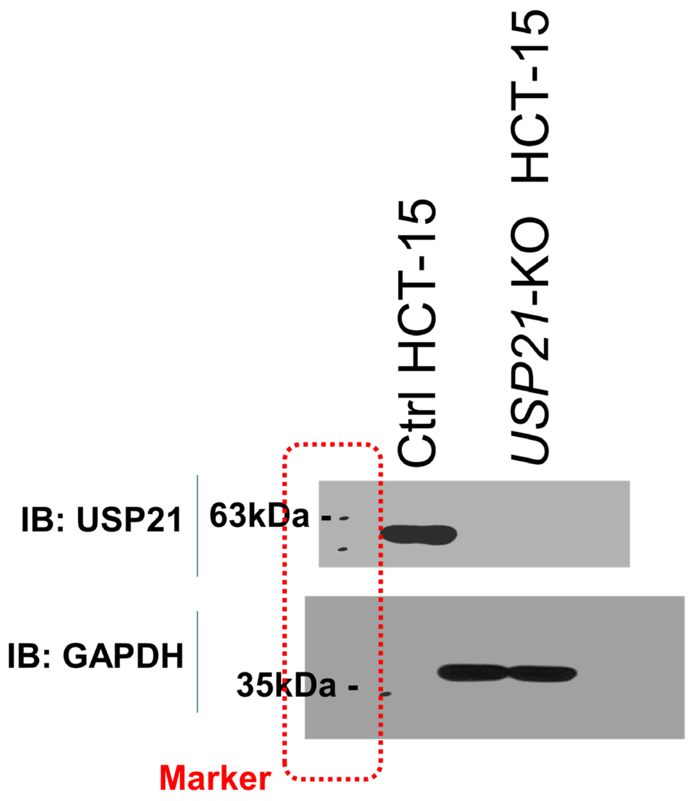

Figure 1H

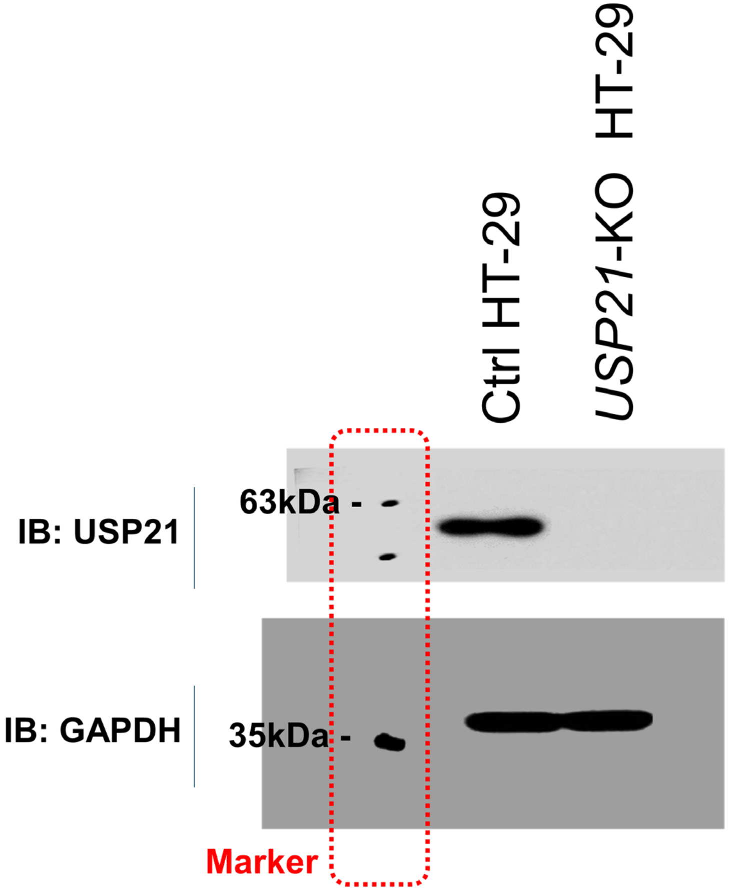

Figure 1I

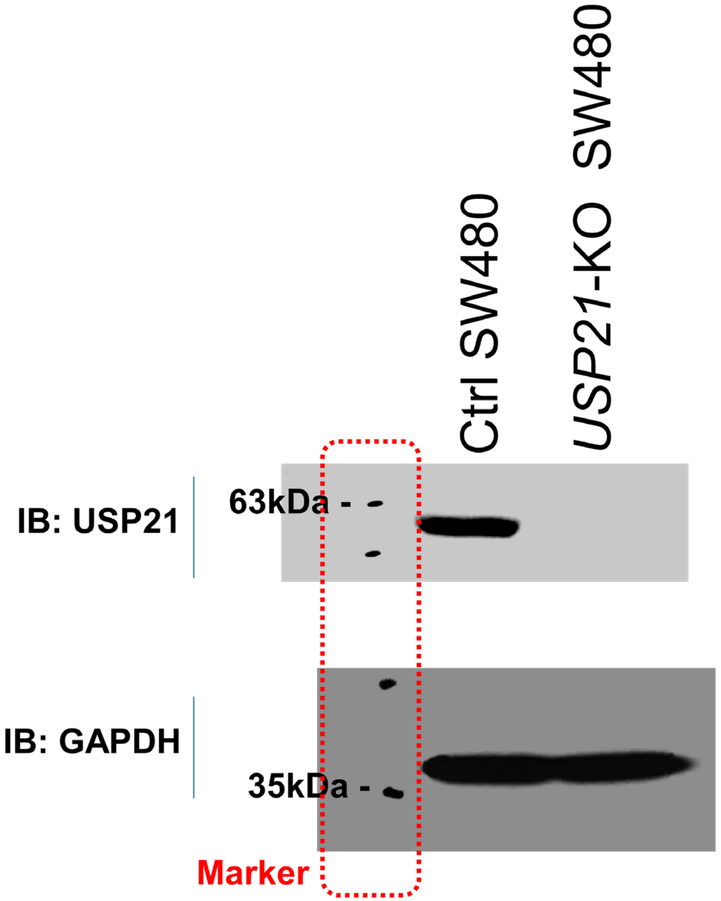

Figure 1J

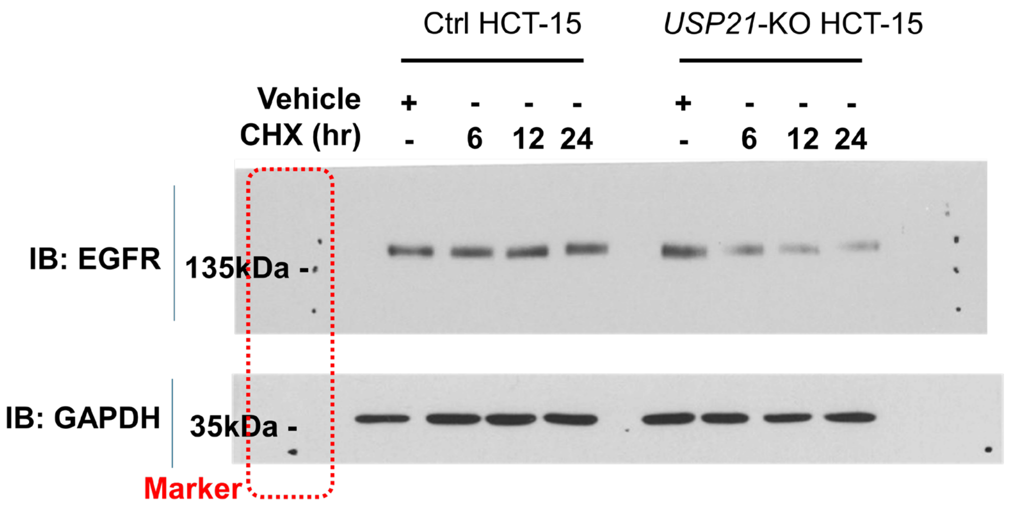

Figure 1K

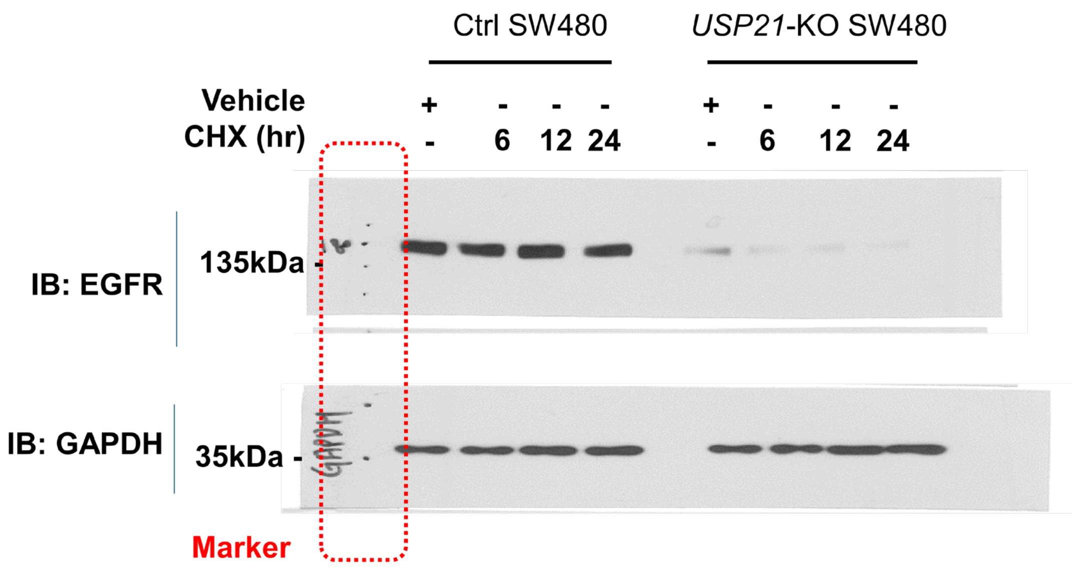

Figure 1L

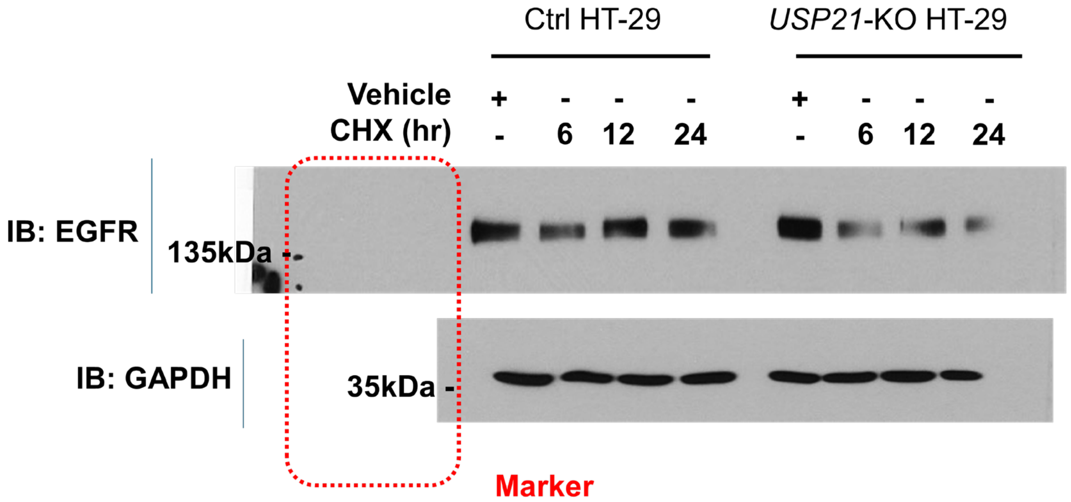

Figure 5B

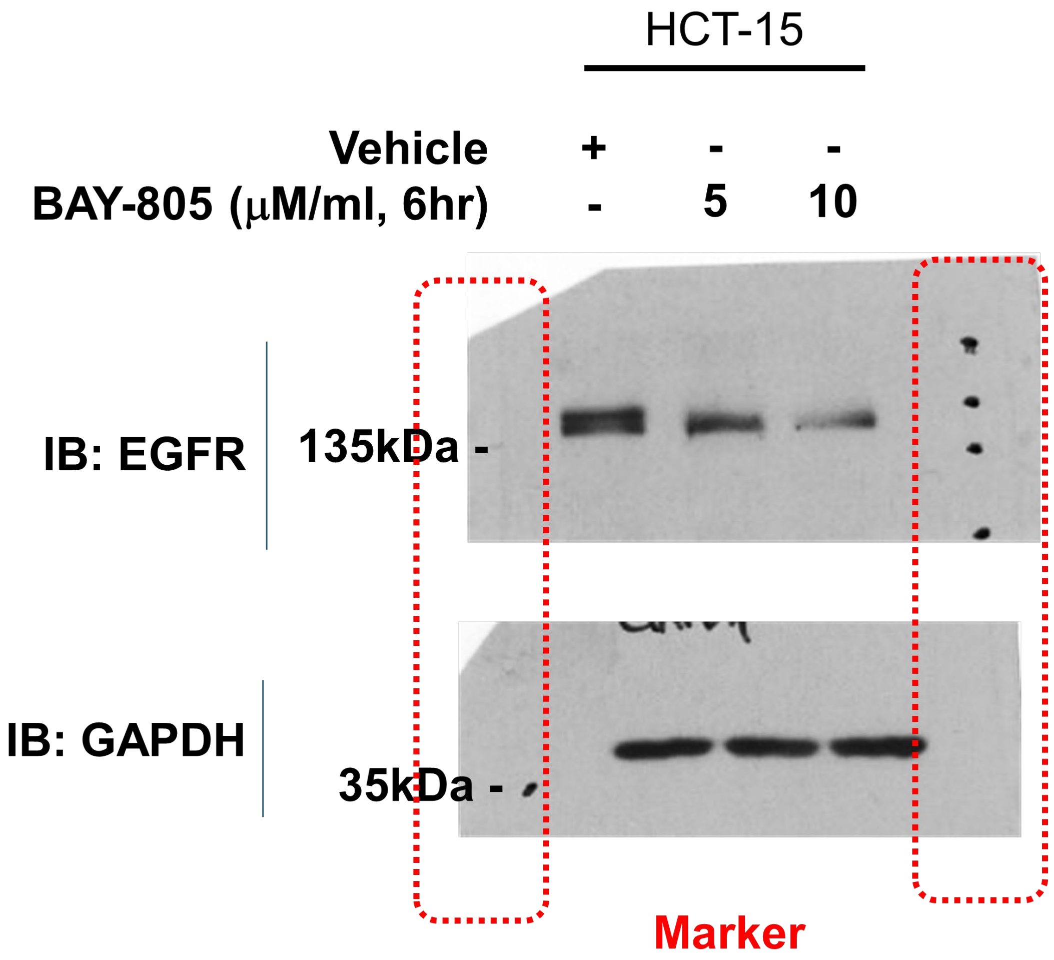

Figure 5D

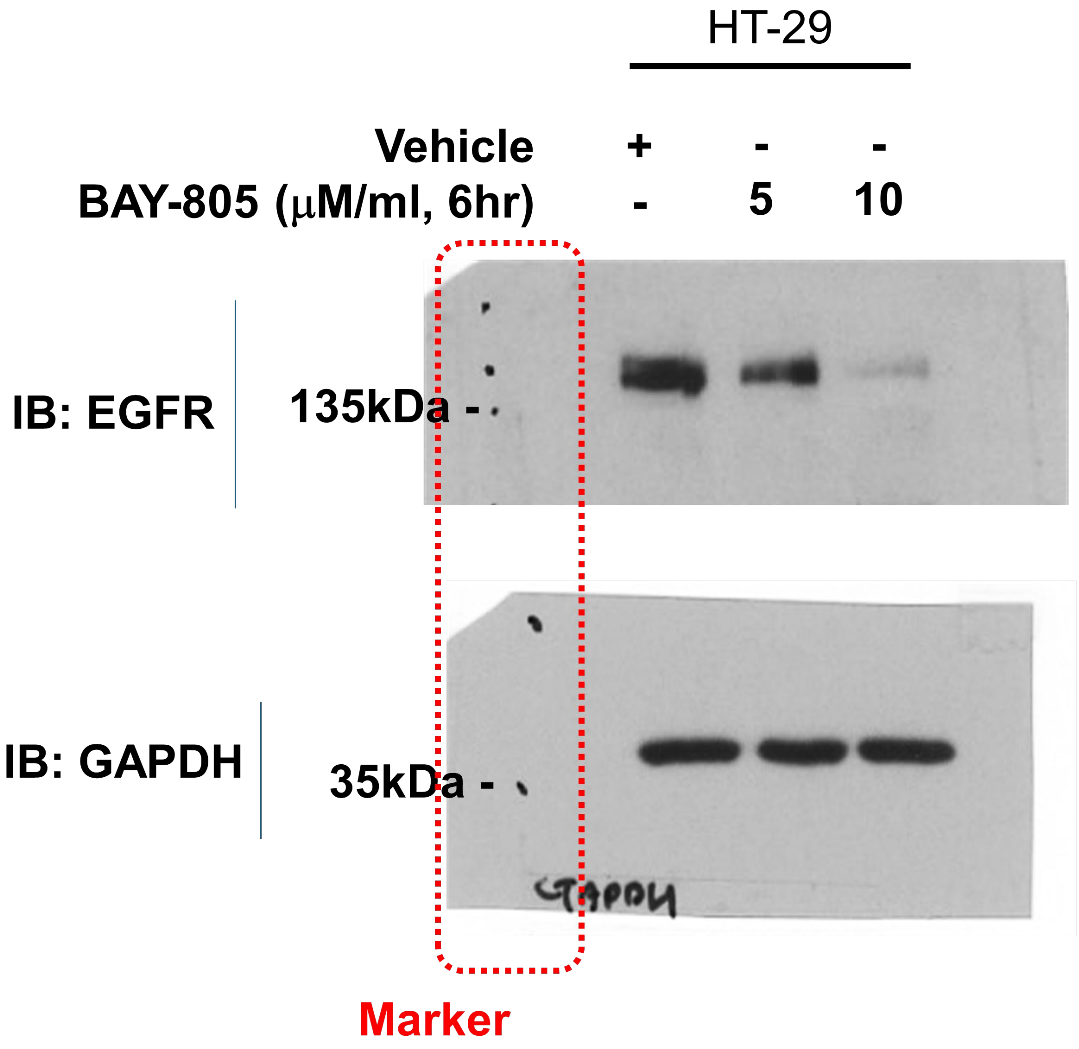

Supplement: Supplementary file 4 — Original data file [file 41420_2024_2255_MOESM4_ESM.pdf]
